# Microglial dectin-1/*Clec7a* in an alzheimer’s disease model: a double-edged sword across the lifespan

**DOI:** 10.3389/fimmu.2026.1864888

**Published:** 2026-09-03

**Authors:** Wen Xi, Ramona M. Rodriguiz, William C. Wetsel, Mari L. Shinohara

**Affiliations:** 1 Department of Integrative Immunobiology, Duke University School of Medicine, Durham, NC, United States; 2 Department of Psychiatry and Behavioral Sciences, Duke University Medical Center, Durham, NC, United States; 3 Mouse Behavioral and Neuroendocrine Analysis Core Facility, Duke University Medical Center, Durham, NC, United States; 4 Department of Cell Biology, Duke University School of Medicine, Durham, NC, United States; 5 Department of Neurobiology, Duke University School of Medicine, Durham, NC, United States; 6 Department of Molecular Genetics and Microbiology, Duke University School of Medicine, Durham, NC, United States

**Keywords:** 5xFAD, alzheimer’s disease, cognitive impairment, dectin-1, fear conditioning, microglia, morris water maze (MWM), mouse behavior assessment

## Abstract

**Introduction:**

Dectin-1, encoded by *Clec7a*, is highly expressed in disease-associated microglia in Alzheimer’s disease (AD), yet its *in vivo* function remains largely unknown.

**Methods:**

We generated *Clec7a*-floxed mice and conditionally deleted dectin-1 in microglia to determine its effects on pathology and behavior using the 5xFAD mice model. No dectin-1 ligand injection was used. Amyloid pathology, immune phenotypes in the brain, and behavior were examined across disease stages.

**Results:**

In 6-month (mo) old mice, microglial dectin-1 reduced dense Aβ plaques in the subiculum without detectable behavioral effects. At 9-mo, microglial dectin-1 impaired memory retention and spatial learning, accompanied by an increased diffuse/dense Aβ plaque ratio in the hilus of the dentate gyrus and limited brain infiltration of Ly6G^lo^ neutrophils. At 16-mo, microglial dectin-1 increased freezing behavior suggestive of heightened emotional reactivity.

**Discussion:**

Microglial dectin-1 exerts age-dependent effects: beneficial for amyloid pathology in mid-life but detrimental for cognition and anxiety-related behavior in later life. These context-specific outcomes highlight the need to consider age, the cell types expressing dectin-1, and dectin-1-activating ligands. Future studies should define the classes of ligands and signaling landscape of microglial dectin-1 across the disease course with the goal of identifying windows where intervention could tilt its function toward sustained neuroprotection.

## 1 Introduction

Dectin-1, encoded by the *Clec7a* gene, is a pattern recognition receptor (PRR) that belongs to the C-type lectin receptor (CLR) family. As the first fungal receptor of its kind to be discovered, dectin-1 has been extensively studied as a β-glucan receptor (1–3). It is widely expressed on the surface of mammalian myeloid cells, mediating anti-fungal immune responses. Upon β-glucan stimulation, dectin-1 activates Card9 through SYK, leading to NF-kB activation (4, 5). For example, in infection by *Candida albicans*, dectin-1 promotes the expression of IL-6, TNF, and IL-23 by dendritic cells, facilitating Th17 adaptive immune responses (6). In addition to pathogen-associated molecular patterns (PAMPs), such as β-glucans, dectin-1 also detects endogenous ligands (7), including but not limited to Galectin-9 (8, 9), annexins (10), vimentin (11, 12), and N-glycans (13). These findings suggest that dectin-1 is a versatile receptor involved in different biological functions beyond antifungal immunity.

Previous studies have shown that dectin-1 signaling can be either beneficial or detrimental during sterile inflammation in the central nervous system (CNS), depending on the context (reviewed in (7)). For example, animal models demonstrated that dectin-1 was detrimental in stroke (14, 15) and spinal cord injury (16), but beneficial in optic nerve injury (17, 18), and experimental autoimmune encephalomyelitis (9). Considering that dectin-1 promotes proinflammatory responses at least in fungal infection settings, it may be counterintuitive for dectin-1 to be protective in CNS inflammatory diseases. In the setting of experimental autoimmune encephalomyelitis (EAE), we found that dectin-1 in myeloid cells promotes production of neuroprotective molecules, particularly oncostatin M (OSM), through a Card9-independent dectin-1 signaling pathway (9). We also found that an endogenous dectin-1 ligand galectin-9 promotes production of OSM by CNS-infiltrated myeloid cells. OSM then signals through the OSM receptor on astrocytes to promote neuroprotection (9).

Although dectin-1 is not detected in microglia under naive conditions (9), its expression is induced in disease-associated microglia (DAM) in human Alzheimer’s disease (AD) and in mouse models of AD (19, 20). More recent studies focusing on SYK, a kinase activated by dectin-1 ligation, demonstrated that intrahippocampal injections of a dectin-1 agonist, pustulan, reduced amyloid-beta (Aβ) plaque burden in 10-week-old 5xFAD mice in a SYK-dependent manner (21). Another study also demonstrated that intraperitoneal injections of a dectin-1 agonist antibody rescued microglia activation in 8-month (mo) old *TREM2^R47H^;5xFAD* mice (22). These mice express a human *TREM2^R47H^* allele associated with high AD risk due to failure of TREM2 to activate SYK (22). Together these studies suggested that SYK activation by dectin-1 ligation limits Aβ plaque burdens and promotes microglia activation. However, it remains unknown whether and how microglia-specific dectin-1 deletion affects the development of AD-like pathology and behavior. In particular, it remains unknown whether and how dectin-1 affects cognitive decline.

In this study, we used a genetic approach to investigate the role of microglial dectin-1 in the 5xFAD mouse model and assessed its impact at different ages. We generated microglia-specific conditional knockout (CKO) of dectin-1. We found that dectin-1 was beneficial in reducing Aβ plaque burdens in 6-mo old mice without detectable changes in the novel object recognition memory (NORM) and Morris water maze (MWM) tests. However, at 9-mo of age, the CKO group outperformed the control group, which retained microglial dectin-1 expression in the MWM test, with the effect being particularly pronounced in females. The CKO group also exhibited better short-term memory in the NORM test, suggesting that microglial dectin-1 was detrimental at this age. Additionally, the CKO group had increased numbers of microglia and neutrophils in the brain, as well as a lower diffuse-to-dense Aβ ratio in the hilus. We also found that neutrophils from 9-mo CKO mice had reduced Ly6G expression. Interestingly, a previous report described that Ly6G^lo^ neutrophils are neuroprotective (18). In mice at 13-mo and older, genotype-dependent histological differences were no longer detected. However, at this age, the CKO group performed better in both short- and long-term memory in a NORM test with lower baseline levels of anxiety, compared to the control mice. These data suggest that microglial dectin-1 limits Aβ deposition in younger mice, but as mice age, microglial dectin-1 becomes detrimental, particularly in mouse behavior.

## 2 Materials and methods

### 2.1 Animals

All mice utilized in this study were on the C57BL/6J 5xFAD background. The following mice were obtained from the Jackson Laboratory (Bar Harbor, ME); C57BL/6J (#000664), 5xFAD (#034848), and *Cx3cr1^CreERT2^* (#020940). The animals were housed in a specific pathogen-free facility at Duke University. The 5xFAD mice were crossed to generate 5xFAD;*Cx3cr1^ERT2Cre^* (“CONT” group) and 5xFAD;*Clec7a^f/f^;Cx3cr1^ERT2Cre^* (“CKO” group, microglia-specific conditional dectin-1 knockout). These mice were heterozygous for both 5xFAD and *Cx3cr1^ERT2Cre^* alleles. All animal experiments were performed as approved by the Institutional Animal Care and Use Committee at Duke University.

### 2.2 Generation of the *Clec7a^f/f^* mouse line

**Figure 1:**
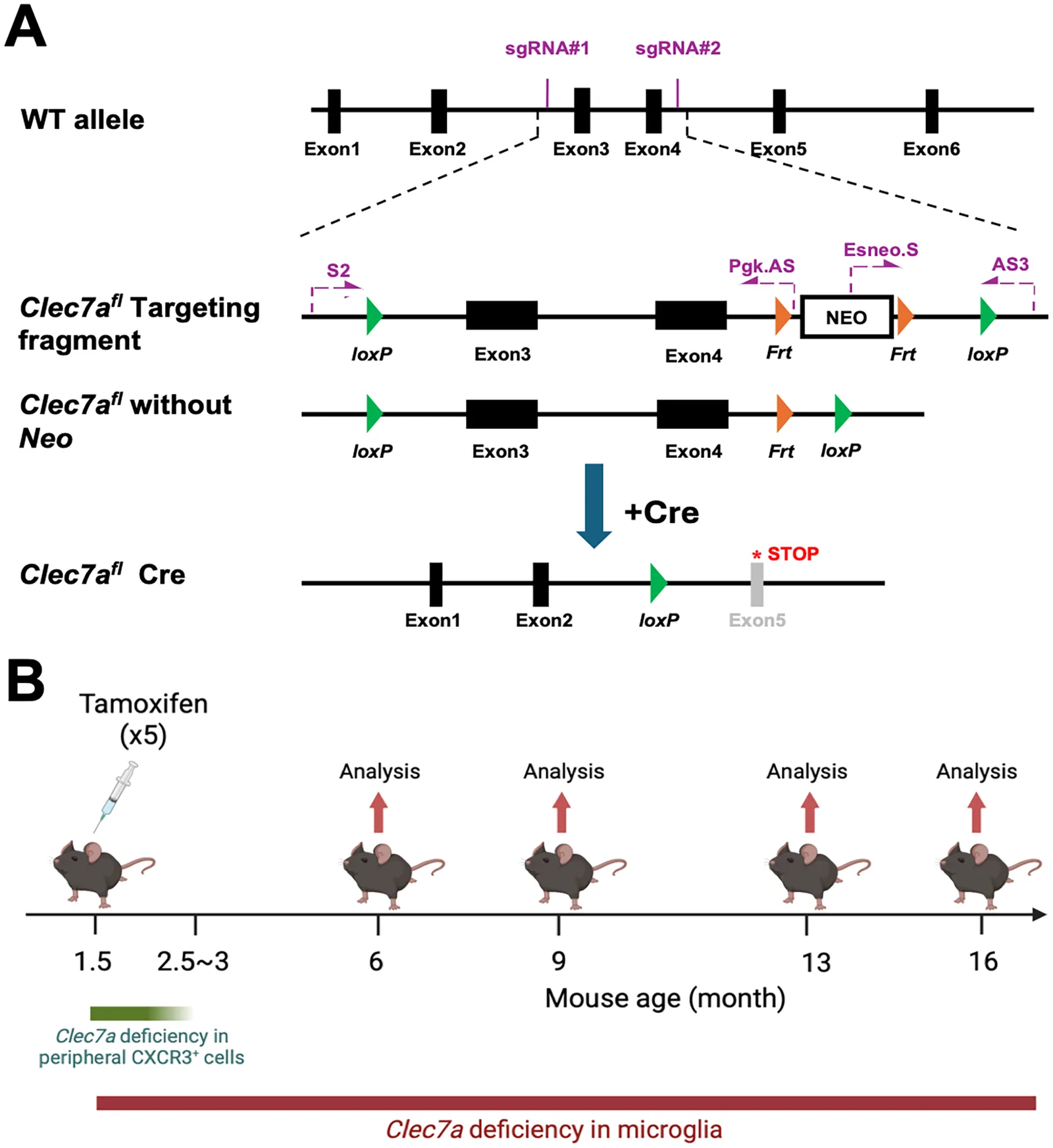
Schematic of the *Clec7a^flox^* allele generation and the timeline of *Clec7a* deletion followed by mouse analysis. **(A)** The *Clec7a^flox^* knock-in allele was engineered using a CRISPR-Cas9 approach with two guide RNAs (sgRNA#1 and sgRNA#2) targeting intronic regions upstream of exon 3 and downstream of exon 4, respectively. The targeting construct introduced *loxP* sites flanking exons 3 and 4 along with a neomycin resistance (NEO) cassette, which was later excised by crossing with *FlpO*-expressing mice. In the absence of Cre recombinase, *Clec7a^flox^* mice express full-length dectin-1. Upon Cre-mediated recombination, exons 3 and 4 were excised, resulting in a frameshift and premature translation termination within exon 5, thereby disrupting dectin-1 expression. The red asterisk in Exon 5 indicates the translation stop position. **(B)** As described in the Methods section, mice were treated with tamoxifen at 6–7 weeks of age (approximately 1.5 months). Tamoxifen treatment induces recombination in all CX3CR1^+^ cell types, resulting in deletion of the *Clec7a* gene. However, within approximately a month after treatment, most peripheral CX3CR1^+^ cells are replaced through hematopoiesis by newly generated bone marrow-derived cells with an intact *Clec7a* gene. In contrast, microglia are not replenished and therefore remain *Clec7a*-deficient throughout the experimental period. Mice were analyzed at 6, 9, 13, and 16 months of age. Illustration was created with BioRender.com.

The *Clec7a^f/f^* mice were created in the Shinohara lab with the assistance of Duke Transgenic Mouse Facility by CRISPR-based homology-independent targeted integration (HITI) (23) in 129/B6N hybrid mouse ES cells. We used two guide RNAs (**Supplementary Table 1**) targeting the upstream intron from exon 3 and the downstream intron from exon 4 (**Figure 1B**). Deletion of exons 3 and 4 resulted in frameshifting in exon 5, introducing a premature stop codon. Allele-specific PCR was performed across both 5’ and 3’ integration junctions, and the ES cell clone, D9, was selected by PCR (**Supplementary Figure 1A**). The clone was further verified by DNA sequencing to confirm the successful integration of the mutant locus. After the germline transmission of the allele, the transgenic line was crossed with *FlpO* mice to remove the neomycin selection marker, thereby establishing the *Clec7a^f/f^* mouse line.

### 2.3 Dectin-1 conditional knockout (CKO)

To achieve microglia-specific *Clec7a* Cre-mediated deletion, we employed a previously published method (24). In brief, 5xFAD;*Clec7a^f/f^;Cx3cr1^ERT2Cre^* mice were intraperitoneally injected with 150 mg/kg tamoxifen once a day over five consecutive days when they were 6 to 7 weeks old. After tamoxifen treatment, peripheral myeloid cells are replaced by newly generated cells through hematopoiesis over approximately 4 weeks. Consequently, these newly derived myeloid cells have never been exposed to tamoxifen, and their *Clec7a* locus remains undeleted. In contrast, microglia are not replenished through hematopoiesis and therefore retain deletion of the *Clec7a* locus (CKO). The 5xFAD;*Cx3cr1^ERT2Cre^* mice, serving as a control group (CONT), were also treated with tamoxifen, as were the CKO mice.

### 2.4 Preparing single-cell suspensions from brains

The mice were euthanized using a gradual-fill CO_2_ method with a displacement rate of 4 liters per minute (approximately 70% of the chamber volume). Animals remained in the chamber for at least 1 min after respiration ceased, after which the mice were transcardially perfused with 10 U/ml heparin in 1X PBS. The brains were isolated, minced, and digested with collagenase D (2 mg/ml) and DNase I (10 μg/ml) in 1X PBS containing 0.5% BSA at 37 °C for 30 minutes. After digestion, single-cell suspensions were prepared and then filtering them through a 70 μm cell strainer. The cells were then resuspended in 38% Percoll in 1X PBS and centrifuged at room temperature at 532 x *g* for 30 minutes without brake. The layer containing lipids and debris was aspirated from the top of the tube, and pelleted brain cells were resuspended in staining buffer. The chemicals and reagents used in this study are listed in **Supplementary Table 2**.

### 2.5 Antibody staining for flow cytometry

Cells were treated with Fc block (CD16/CD32) before being stained with fluorophore-labeled antibodies listed in **Supplementary Table 2**. The gating strategy is illustrated in **Supplementary Figure 2**. Flow cytometry was performed using either a BD Fortessa X-20 or a Cytek Aurora flow cytometer, and the data were analyzed using FlowJo Software version 10 (FlowJo LLC).

### 2.6 *In vivo* phagocytosis

Mice received an *intraperitoneal (i.p.)* injection of 1 mg/kg of Methoxy-x04 in 1X PBS. Two hours later, brains were collected to assess microglial uptake of Methoxy-X04-labeled Aβ by flow cytometry. Microglia that had phagocytosed Aβ were identified as Methoxy-x04-positive cells within the CD11b^+^CD45^lo^ microglia population.

### 2.7 Immunofluorescent staining and microscopic imaging

Brains were removed and immediately fixed in 4% paraformaldehyde (PFA) for one day. The fixed brains were dehydrated in 30% sucrose for 72 hours until they sank to the bottom. Then, they were embedded in Tissue-Tek O.C.T. compound (Sakura) and snap-frozen with liquid nitrogen. Coronal brain sections were collected approximately 1.5 mm posterior to the bregma, and sagittal sections were taken about 0.5 mm lateral to the midline. The sections were permeabilized with 0.1% Triton X-100 in TBS for 30 minutes and blocked for one hour using 2% Bovine Serum Albumin (BSA) in 1x TBS. Staining was performed on floating brain sections with a primary antibody in 2% BSA/1x TBS-T overnight at 4 °C, followed by a secondary antibody for one hour at room temperature. Primary and secondary antibodies used are listed in **Supplementary Table 2**. Methoxy-X04 (1 µM) staining was carried out for one hour at room temperature. The stained brain sections were imaged using a Leica SP8 confocal microscope or an Andor Dragonfly Spinning Disk Confocal microscope. The images were analyzed with the Fiji package of ImageJ.

### 2.8 Behavioral tests

The behavioral tests were conducted at the Duke Mouse Behavioral and Neuroendocrine Analysis Core Facility as previously described (25). Before each test, we performed baseline assessments to ensure all mice had normal hearing, vision, and mobility.

*Novel Object Recognition Memory (NORM) test* consisted of a training phase and two retention tests (26). In the training phase, mice were habituated to the test chamber for 30 minutes. Subsequently, two identical objects were placed in opposite corners of the chamber without disturbing the animal. The mouse was given 10 minutes to explore the objects, then returned to its home cage until retention testing at 20 minutes for short-term (STM) and 24 hours long-term memory (LTM). Briefly, one of the 2 identical objects from training was replaced with a novel object of approximately the same size and height for STM and this novel object was replaced with another novel object for LTM testing. In between animals or tests, the test chamber and objects are cleaned with an odorless disinfectant to remove any odor cues. All behaviors were video-recorded and the response (time with each and total time with both objects, contacts with both objects, and distance traveled) were automatically analyzed by EthoVision XT 18 software (Noldus Information Technologies, Leesburg, VA). Preference for the novel object was calculated as the time exploring the novel object minus the time exploring the familiar object, divided by the total time exploring both objects. Longitudinal assessments were performed on mice at 6, 9, and 13 months of age.

*Morris Water Maze (MWM) Test* was conducted in a circular pool of water, made opaque by the addition of a nontoxic white dye (26). The pool was divided into quadrants: northeast (NE), southeast (SE), northwest (NW), and southwest (SW). Spatial cues were placed on the walls of the test room to aid the mice in navigation. One quadrant was designated as the location of the hidden platform, which was placed 1 to 2 cm below the water surface, and it was large enough for the mouse to stand with all four feet. The day before testing, mice were trained to sit on the platform for 30 seconds and allowed to swim freely around the maze for 60–90 seconds before being guided to the platform. On test days, individual mice were released from different locations around the perimeter of the pool and allowed to swim until they found the platform or 60 seconds elapsed. On probe trial days, mice were given a subsequent separate 60-second trial in which the hidden platform had been removed. These acquisition trials were conducted over 8 consecutive days and were followed by 8 days during which the position of the hidden platform was reversed and moved to the SW quadrant. During both acquisition and reversal testing, probe trials were conducted every other day of testing (e.g., 2, 4, 6, 8, 10, 12, 14, 16). Performance in the MWM was assessed based on swim distance or path length (cm) and swim time (sec) required to reach the hidden platform. For probe trials, the same metrics were applied: the time mice swam in the target quadrant relative to other quadrants was used to calculate their zone preferences. To avoid a learning-to-learn effect, each cohort was evaluated on a single MWM test. Assessments were performed with mice at 6 and 9 months of age, but not longitudinally.

*Fear Conditioning (FC) Test* consisted of three sessions: conditioning, contextual, and cued fear testing, which were examined over three consecutive days (27). During all sessions, all incidences of spontaneous freezing behaviors were recorded. Due to the long-term behavioral changes resulting from the fearful experience, the FC test was conducted only after all other behavioral tests were completed. In the conditioning session on day 1, mice were placed into the apparatus and allowed to explore the chamber for 2 minutes. Subsequently, the mouse was presented with a conditioned stimulus (CS), a 30-second 65 dB, 2.9 kHz tone, that terminated simultaneously with a 2-second, 0.4 mA scrambled foot-shock as the unconditioned stimulus (UCS). Following this noxious experience, the mouse remained in the conditioning chamber for an additional 30 seconds before being returned to its home cage. Twenty-four hours later, contextual fear was assessed in the absence of the CS and UCS. Here, the mouse was returned to the same test chamber and given free access to the apparatus for 5 minutes in the absence of tone or foot shock. The next day, cued fear was evaluated in the presence of the CS. The mouse was placed in a test chamber where the texture of the floor, the patterns and colors of the walls, and the space within the chamber differed from those during conditioning. After a 3-minute period of free exploration, the CS was presented continually for 2 minutes. Freezing behavior was scored and expressed as the total time (seconds) the mouse was immobile per minute during the 5 min test. The same cohort of mice, tested for NORM, was used at 16-mo old.

### 2.9 Statistical analysis

The statistical analysis were performed with IBM SPSS Statistics 29 programs (IBM, Chicago, IL). The data are presented as means and standard errors of the mean (SEMs). The NORM, MWM, and fear conditioning data were analyzed by repeated-measures ANOVA (RMANOVA), followed by Bonferroni corrected pair-wise comparisons. All other statistical analysis were run using GraphPad Prism software (version 9 or 10). A *p<0.05* was considered significant. All results were plotted using GraphPad Prism 10.4.1.

## 3 Results

### 3.1 Generation of *Clec7a*-*flox* mice

To achieve cell type-specific dectin-1 deletion, we first generated a *Clec7a^f/f^* mouse line by targeting and removing a region of the *Clec7a* locus that includes exons 3 and 4 (**Figure 1A**), using the CRISPR-Cas9 system. This introduced Cre-mediated deletion of the targeted region and a premature STOP codon in exon 5. The successful incorporation of the floxed exons in the embryonic stem cells was validated by PCR, confirming the presence of 5’ and 3’ *loxP* sites, as well as the *Frt*-flanked Neo gene, in the ES cell clone D9 (**Supplementary Figure 1A**). We further sequenced the regions for nucleotide-level confirmation. The *Clec7a^f/f^* founder mice were crossed to generate 5xFAD;*Clec7a^f/f^;Cx3cr1^ERT2Cre^* mice. Since CX3CR1 is expressed not only on microglia but also on certain subsets of peripheral myeloid cells, we employed a tamoxifen “wash-out” approach as previously described (24). In this approach, we treated the CKO mice with repeated tamoxifen injections. The period of 4 weeks after tamoxifen treatment allows hematopoiesis to repopulate myeloid cells, whereas microglia have a minimal turnover and do not repopulate after this treatment (24). These 5xFAD;*Clec7a^f/f^;Cx3cr1^ERT2Cre^* mice that went through the tamoxifen treatment were conditional knockout (CKO) of dectin-1 in microglia (**Figure 1B**). Tamoxifen-treated 5xFAD;*Cx3cr1^ERT2Cr^*^e^ mice were used as a control group (CONT). All mice were heterozygous for the *Cx3cr1^CreERT2^* allele (*Cx3cr1^CreERT2/+^*) and retained one wild-type *Cx3cr1* allele. Thus, CONT and CKO mice had equivalent *Cx3cr1* gene dosage, despite disruption of one *Cx3cr1* allele. To confirm CKO, flow cytometry and immunofluorescent (IF) microscopy were performed. The loss of dectin-1 expression was observed in microglia (CD45^lo^CD11b^+^) but not in brain-infiltrated myeloid cells (CD45^hi^CD11b^+^) in CKO mice (**Supplementary Figures 1B, C**). IF images also indicated the absence of dectin-1 expression on Iba-1^+^ cells in the proximity of an Aβ plaque from CKO, but not CONT mice (**Supplementary Figure 1D**).

### 3.2 Microglial dectin-1 reduces dense Aβ in 6-mo old mice

**Figure 2:**
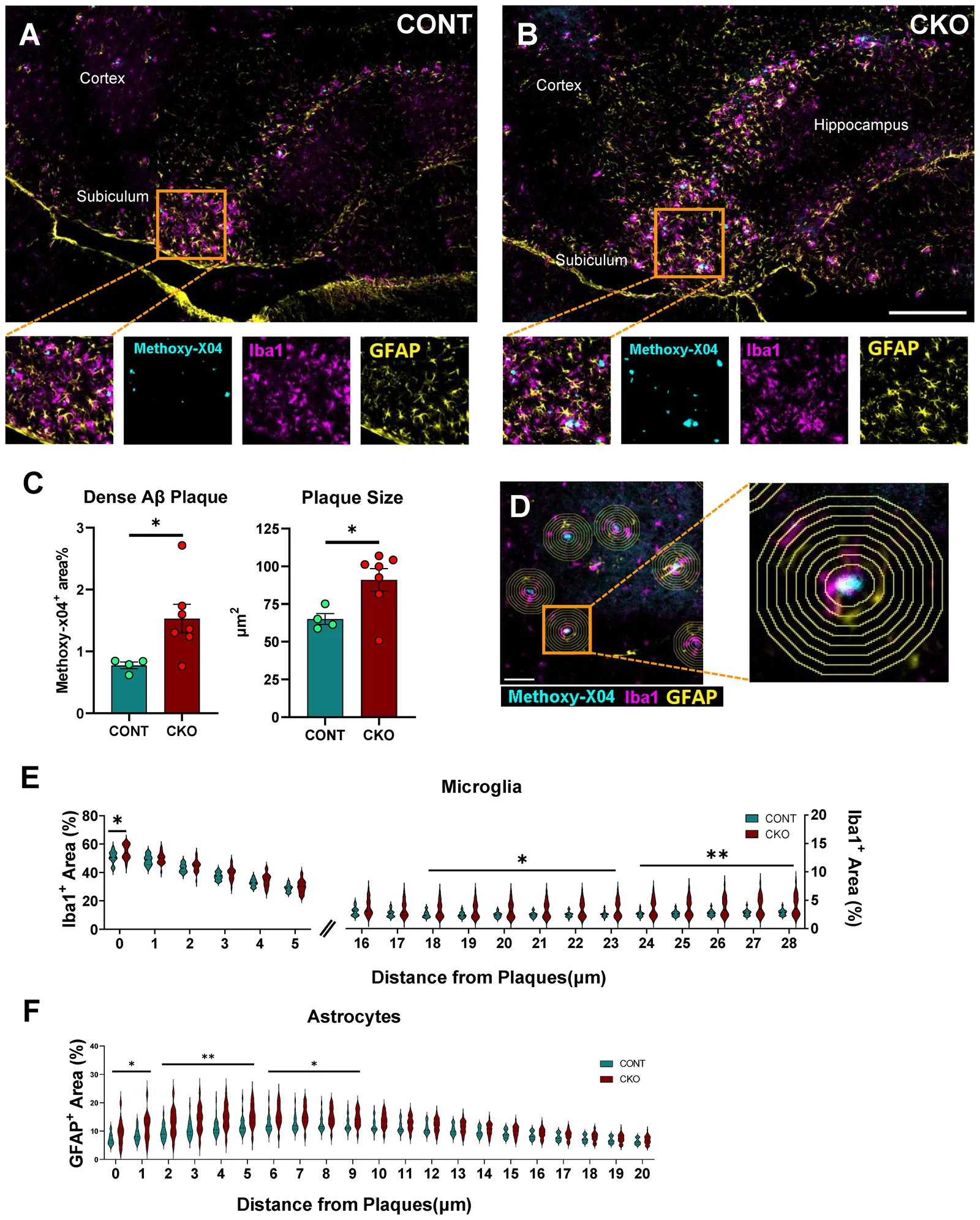
Evaluation of Aβ plaques, microglia, and astrocytes in 6-month-old mice. **(A, B)** Representative images of sagittal hippocampal sections from 6-mo old 5xFAD CONT (A) and CKO (B) mice. Orange squares denote the subiculum. Immunofluorescence staining visualized Methoxy-X04 (dense Aβ plaques), Iba1, and GFAP (astrocytes). Based on signal distribution and limited infiltration of peripheral myeloid cells at this age, Iba1^+^ cells are primarily considered microglia. Scale bar, 100 μm. **(C)** Quantification of Methoxy-X04^+^ plaque burden in the subiculum, which fits within a 350-μm diameter circle for evaluation. Methoxy-X04^+^ area (%) and average plaque size were measured. Each data point denotes a value from one mouse. **(D–F)** Spatial distribution analysis of glial cells around Aβ plaques in the cortex. Concentric rings of 1-μm width were generated from plaque edges **(D)** to quantify Iba1^+^ **(E)** and GFAP^+^ **(F)** signals. “0 μm” represents the first 1-μm ring adjacent to the plaque edge. Data reflect average values per mouse from all plaques in the cortex (CONT, *n=6*; CKO, *n=9*). Scale bar, 50 μm. Data are presented as mean ± SEM. *p < 0.05, **p < 0.01 by two-tailed unpaired Student’s t-test.

To evaluate the impact of dectin-1 on microglia, we examined the areas and sizes of Aβ plaques in 6-mo old mice using Methoxy-X04 staining, along with co-staining of Iba1 and GFAP. Given that the hippocampal subiculum contains dense Aβ plaques, we quantitatively assessed that region (**Figures 2A, B**). The CONT group exhibited a significantly lower Methoxy-X04-positive plaque area and smaller average plaque size than the CKO group (**Figure 2C**). Conversely, Aβ plaques were still sparsely distributed in the cerebral cortex (**Supplementary Figure 3A**), relative to the subiculum, with no significant differences noted between the CKO and CONT groups across five separate regions in the cortex (**Supplementary Figure 3B**). These findings suggest a beneficial role for dectin-1 in microglia, contributing to the reduction of Aβ accumulation, particularly in the subiculum of mice aged approximately 6 months.

### 3.3 Microglial dectin-1 is associated with altered plaque-centered glial distribution in the cortex at 6-mo

We evaluated the distribution of glial cells. Since glial cells were too dense in the subiculum for this analysis, the cortex, where plaques and surrounding glial signals were more clearly separated, was analyzed instead. Iba1 and GFAP fluorescent signals were evaluated within concentric 1μm-wide rings around the edges of each Aβ plaque (**Figure 2D**). Microglia were tightly clustered around plaques, and their densities decreased with increasing distance from the plaques (**Figure 2E**). The CKO group showed greater Iba1-positive area 18–28 μm from plaque edges and greater GFAP-positive area within 9 μm of plaque edges (**Figures 2E, F**).

The percentages of areas with Iba-1-positive signals (**Supplementary Figure 3C**) and GFAP-positive signals, as well as astrocyte sizes (**Supplementary Figure 3D**), were not statistically different between the CONT and CKO groups. In the subiculum, glial cell frequencies were also similar between the two groups (**Supplementary Figures 3E, F**).

In summary, microglial dectin-1 was associated with differences in the plaque-centered spatial distribution of microglia and astrocytes in the cortex, despite the absence of a detectable genotype-dependent difference in cortical Aβ plaque burden. Because this analysis could not be reliably performed in the densely affected subiculum, these cortical spatial changes should not be interpreted as explaining the dectin-1-dependent plaque phenotype observed in the subiculum.

### 3.4 No cognitive impact by microglia-specific dectin-1 deletion in 6-mo old mice

We assessed cognitive functions in 6-mo old mice using the Novel Object Recognition Memory (NORM) test. The CKO group showed a trend toward reduced preference for the novel object in both STM and LTM testing; however, no significant differences were observed between CONT and CKO mice (**Supplementary Figure 4A**). Similarly, in the MWM test, both groups performed comparably during acquisition and reversal trials (**Supplementary Figure 4B**). These findings suggest that despite the observed microglial dectin-1-mediated effects on Aβ plaques in the subiculum, 6-mo old 5xFAD mice may still be at an early disease stage insufficient to manifest detectable cognitive impairments.

### 3.5 Local decrease of diffuse/dense Aβ plaque ratios and altered immune cell brain infiltration in CKO mice at 9-mo of age

**Figure 3:**
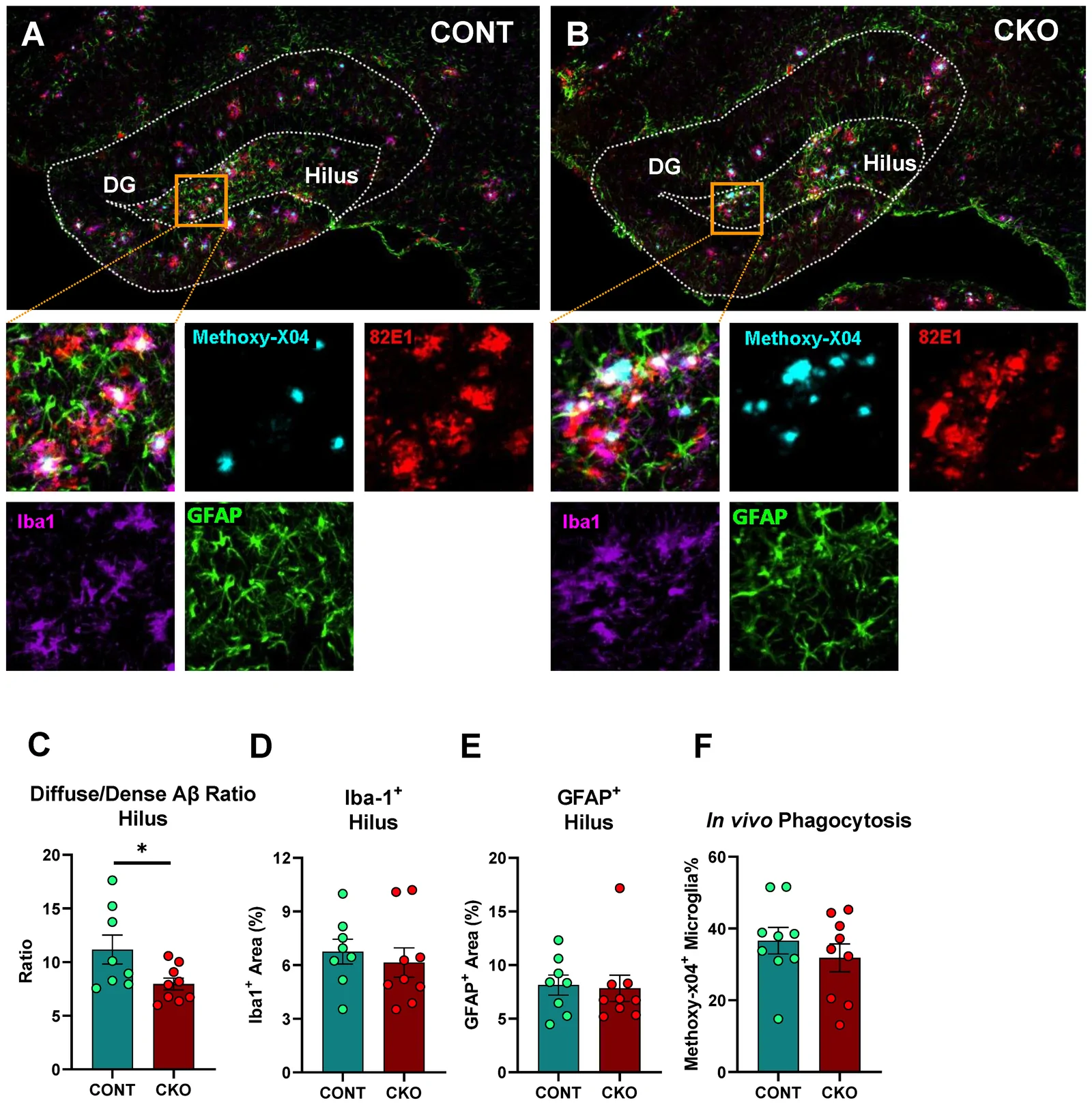
Evaluation of Aβ plaques, glial cells, and microglial phagocytosis in 9-month-old mice. **(A, B)** Representative coronal hippocampal sections from the dentate gyrus (DG) of 5xFAD CONT **(A)** and CKO **(B)** mice. Immunofluorescence staining visualized Methoxy-X04 (dense Aβ plaques), 82E1 antibody (diffuse Aβ), Iba1 (primarily microglia), and GFAP (astrocytes). Scale bar, 300 μm. **(C)** Quantification of the diffuse-to-dense Aβ area ratio within the hilus. *p < 0.05 by two-tailed unpaired Student’s t-test. **(D, E)** Quantification Iba1-positive **(D)** and GFAP-positive **(E)** areas within the hilus. **(F)** *In vivo* Aβ phagocytosis by microglia, assessed two hours after intraperitoneal injection of Methoxy-X04 (1 mg/kg), using flow cytometry to detect Methoxy-X04^+^ microglia. Each data point denotes a value from one mouse. Data represented as mean ± SEM.

We analyzed brain pathology in 9-mo old mice, focusing on the dentate gyrus (DG), where both CONT and CKO mice showed substantial Aβ plaque burden (**Figures 3A, B**). Dense Aβ plaques were visualized using Methoxy-X04, and diffuse Aβ with the 82E1 monoclonal antibody, alongside Iba-1 and GFAP to assess glial responses. Quantitative analysis of the entire DG showed no statistically significant differences in dense or diffuse Aβ burden (**Supplementary Figure 5A**). Although diffuse-to-dense Aβ ratio tended to be lower in CKO mice, the difference was not statistically significant (**Supplementary Figure 5B**). Iba-1^+^ and GFAP^+^ areas were comparable between CONT and CKO mice (**Supplementary Figure 5C**).

Before quantitatively comparing CONT and CKO mice, we further selected the hilus of DG because plaques and glial signals were abundant yet sufficiently separated for consistent quantification. The hilus serves as a critical relay zone between granule cells and CA3 pyramidal neurons, integrating inputs and modulating signal propagation through the hippocampus. Although areas of diffuse and dense Aβ were comparable between groups (**Supplementary Figure 5B**), the diffuse-to-dense Aβ plaque ratio was higher in CONT than in CKO in the hilus (**Figure 3C**). Differences were not observed in Iba-1^+^ (**Figure 3D**) and GFAP^+^ areas (**Figure 3E**) in the hilus.

To assess Aβ uptake *in vivo*, Methoxy-X04 was intraperitoneally administered, and Methoxy-X04^+^ microglia (CD11b^+^CD45^lo^) from the whole brain were quantified by flow cytometry two hours later. No difference in Aβ uptake by microglia was observed between CONT and CKO mice (**Figure 3F**).

These data indicated that, at 9-mo of age, microglial dectin-1 expression in CONT mice was associated with a higher diffuse-to-dense Aβ area ratio in the hilus. This difference may reflect altered local plaque organization, but the outcome was not accompanied by a detectable difference in *in vivo* microglial Aβ uptake. Considering the beneficial role of dectin-1 at 6-mo old, these findings suggest that the effects of microglial Dectin-1 may shift between 6 and 9 months of age.

### 3.6 Microglial dectin-1 deficiency alters the immune cell landscape in the brain at 9-mo old

**Figure 4:**
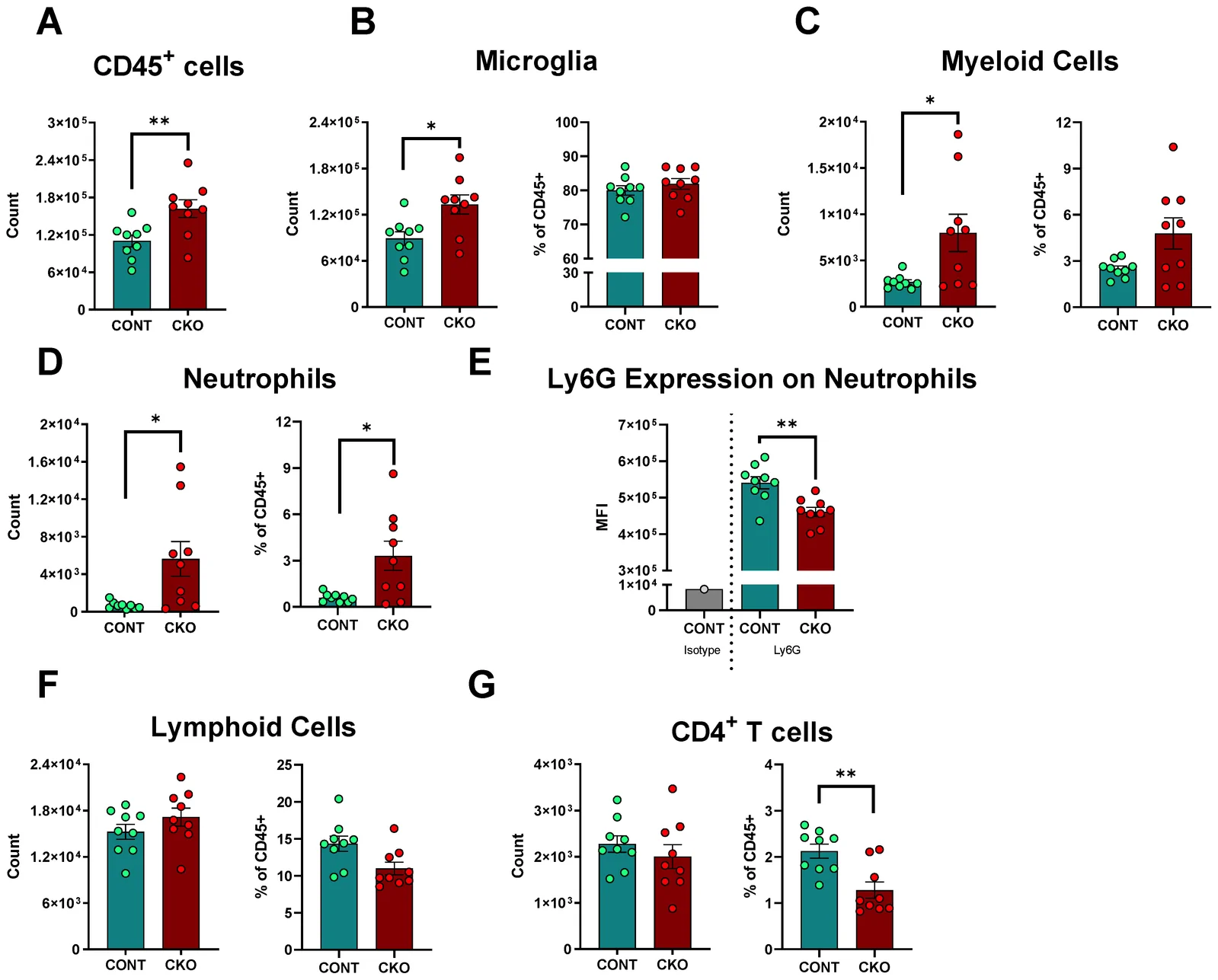
Populations of brain-infiltrated leukocytes in 9-mo old mice. Numbers and proportions (within CD45^+^ cells) of indicated immune cell populations were compared between CONT and CKO mice at 9 months of age. **(A)** Total CD45^+^ cell counts. **(B–D)** Counts and proportions of microglia **(B)**, brain-infiltrating myeloid cells excluding microglia **(C)**, and neutrophils **(D)**. **(E)** Ly6G staining MFI values of neutrophils. **(F, G)** Counts and proportions of lymphocytes **(F)**, and CD4^+^ T cells **(G)**. Each data point denotes a value from one mouse. Data represented as mean ± SEM. *p < 0.05; **p < 0.01 by two-tailed unpaired Student’s t-test.

We assessed immune cell populations in 9-mo old CONT and CKO mice by flow cytometry (**Supplementary Figure 2**). Total CD45^+^ cells, including microglia, were maintained in CKO brains but significantly reduced in CONT mice (**Figure 4A**), primarily due to decreased microglial numbers in the CONT group (**Figure 4B**). In contrast, brain-infiltrated myeloid cells were significantly increased in the CKO group compared to CONT group (**Figure 4C**). In particular, neutrophil numbers and frequencies were significantly elevated in CKO mouse brains (**Figure 4D**) with decreased Ly6G expression levels (**Figure 4E**). A previous study demonstrated neuroprotective roles of Ly6G^lo^ neutrophils (18). It is possible that the absence of microglial dectin-1 promotes recruitment or accumulation of the Ly6G^lo^ neutrophil subpopulation.

Although total lymphocyte infiltration did not differ significantly (**Figure 4F**), the CONT group exhibited a significant increase in CD4^+^ T cells (**Figure 4G**). Other immune cell types, including B cells (**Supplementary Figure 5E**) and CD8^+^ T cells (**Supplementary Figure 5F**), showed no differences between CONT and CKO groups. These findings suggest that microglial dectin-1 influences the brain’s immune milieu, especially by limiting recruitment of neutrophils, possibly a neuroprotective Ly6G^lo^ subset.

### 3.7 Microglial dectin-1 impairs cognitive memory retention at 9- and 13-mo

**Figure 5:**
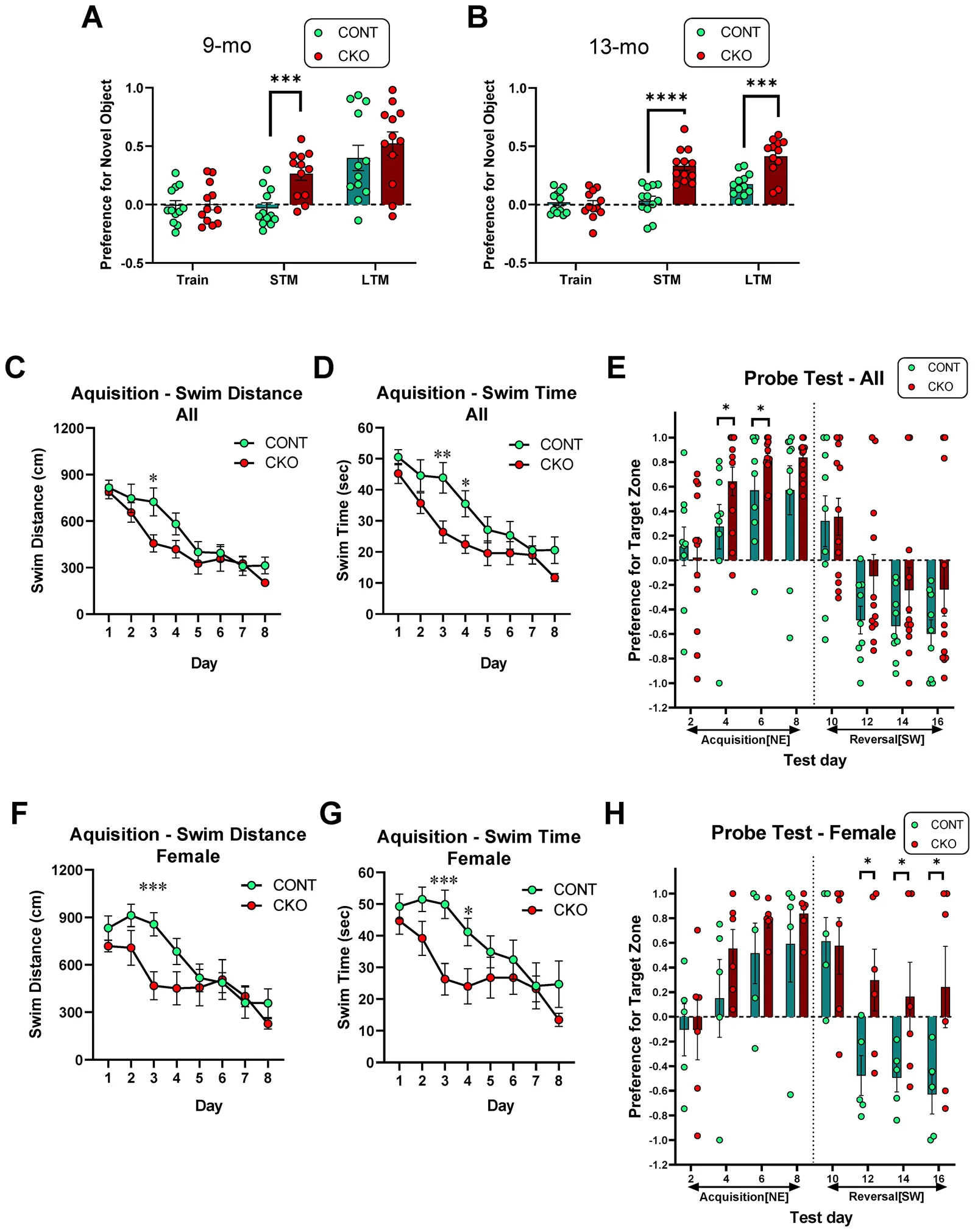
Performance in the novel object recognition memory (NORM) and morris water maze (MWM) tests in aging mice. **(A, B)** NORM tests of 5xFAD CONT and CKO mice at 9-mo **(A)** and 13-mo **(B)**. Short-term memory (STM) and long-term memory (LTM) were assessed 20 minutes and 24 hours after training, respectively. Each data point denotes a value from one mouse. **(C, D)** MWM acquisition phase using 9-mo old male and female mice. Swim distance **(C)** and swim time **(D)** were recorded across trials. Each data point denotes the group average (CONT, *n=9*; CKO, *n=12*). **(E)** Probe trial testing conducted during acquisition and reversal on “even” days of acquisition and reversal without the platform. During acquisition the target zone was the Northeast (NE) quadrant and during reversal the target was the Southwest (SW) quadrant. Swim time spent in each target zone was quantified. Each data point denotes a value from one mouse. **(F–H)** Data in panels **(C–E)** reanalyzed to include only female mice. Each data point denotes the group average (CONT, *n=5*; CKO, *n=6*) **(F, G)**. Each point denotes a value from one mouse **(H)**. Data are presented as mean ± SEM. *p < 0.05; **p < 0.01; ***p < 0.001; ****p < 0.0001 by repeated measures ANOVA with Bonferroni corrected pair-wise comparisons.

We assessed cognitive performance using the NORM test in 9- and 13-mo old mice from the same cohort previously tested at 6-mo old (**Supplementary Figure 4**). At 9-mo old, CONT mice exhibited significantly impaired short-term memory (STM) compared to CKO mice, while long-term memory (LTM) was comparable between groups (**Figure 5A**). At 13 months, CONT mice showed lower STM and LTM performance than CKO mice (**Figure 5B**). Control measures, including contact duration and frequency of contact with objects and total distance traveled, did not differ between groups (**Supplementary Figures 6A, B**), indicating that decreased novel object preference in CONT mice reflects impaired memory retention relative to CKO mice (**Figures 5A, B**). In other words, the presence of microglial dectin-1 was detrimental in NORM at 9- and 13-mo.

To examine possible sex differences, we analyzed STM and LTM separately for females (**Supplementary Figures 6C, D**) and males (**Supplementary Figures 6E, F**) across all three ages, 6-, 9-, and 13-mo old. Although no statistical changes were observed in 6-mo old mice, both sexes in the CONT group displayed a pronounced STM decline at 9 and 13 months, while CKO mice retained STM (**Supplementary Figures 6C, E**). The LTM decline at 13 months, however, was observed only in male CONT mice (**Supplementary Figure 6F**).

These findings suggest that microglial dectin-1 impairs memory retention, with the STM decline emerging by 9-mo and LTM impairment by 13-mo, particularly in males, highlighting an age- and sex-dependent detrimental effect of microglial dectin-1.

### 3.8 Microglial dectin-1 impairs spatial learning and modulates cognitive flexibility in a sex-specific manner

We conducted the Morris Water Maze (MWM) test to assess spatial learning, retention, and cognitive flexibility with 9-mo old mice. When data from both sexes were combined, CONT mice—with the presence of microglial dectin-1— showed longer acquisition swim distances on day 3 (**Figure 5C**) and swim times on day 3 and 4 (**Figure 5D**) compared to CKO mice, indicating delayed learning. Reversal testing, where the platform was relocated to the SW zone, revealed no significant group differences (**Supplementary Figures 7A, B**). In the probe trial without the platform, CKO mice spent more time in the target (NE) zone, consistent with better memory retention (**Figure 5E**).

Sex-stratified analysis revealed that female CONT mice exhibited impaired spatial learning, with significantly greater swim distances and times during acquisition compared to CKO females (**Figures 5F, G**). Although the trend did not reach significance, CONT females showed slightly better reversal performance (**Supplementary Figures 7C, D**). Interestingly, in the probe trial during the reversal testing, CKO females retained a stronger preference for the original NE zone, consistent with reduced cognitive flexibility (**Figure 5H**). This implies that microglial dectin-1 may support cognitive flexibility at least in females, although it impairs learning during the acquisition phase. Male mice, by contrast, showed no differences in spatial learning: acquisition and reversal performance, and probe trial performance (**Supplementary Figures 7E–I**). Notably, all males—regardless of genotype—quickly adapted to the new target SW zone on day 10 in the probe trial (**Supplementary Figure 7G**).

In summary, microglial dectin-1 exerts sex-dependent effects on spatial learning and memory at 9-mo old. In female mice, it impairs learning during acquisition, while its absence compromises cognitive flexibility by promoting the persistence of outdated spatial information.

### 3.9 No anxiety-related difference observed in CKO mice at 9-mo old

To evaluate emotional memory and baseline anxiety, we conducted fear conditioning on 9-mo old mice (25). On day 1, mice explored the conditioning chamber for 2 minutes before presentation of a 30-second tone (Conditioned stimulus, CS) paired with a 0.4 mA foot-shock (Unconditioned stimulus, UCS) in the final 2 seconds of conditioning (**Supplementary Figure 7H**), and mice remained in the chamber for 30 seconds. On day 2 (context test), mice were returned to the conditioning chamber without CS or UCS (**Supplementary Figure 7I**). On day 3 (cued test), they were placed into a novel chamber for 3 minutes without the CS and UCS; this was followed with continual CS for 2 minutes after entry (**Supplementary Figure 7J**). Both groups of mice performed similarly at all time points in the test.

### 3.10 No histological or immunophenotypic differences observed in aged mice lacking microglial dectin-1

**Figure 6:**
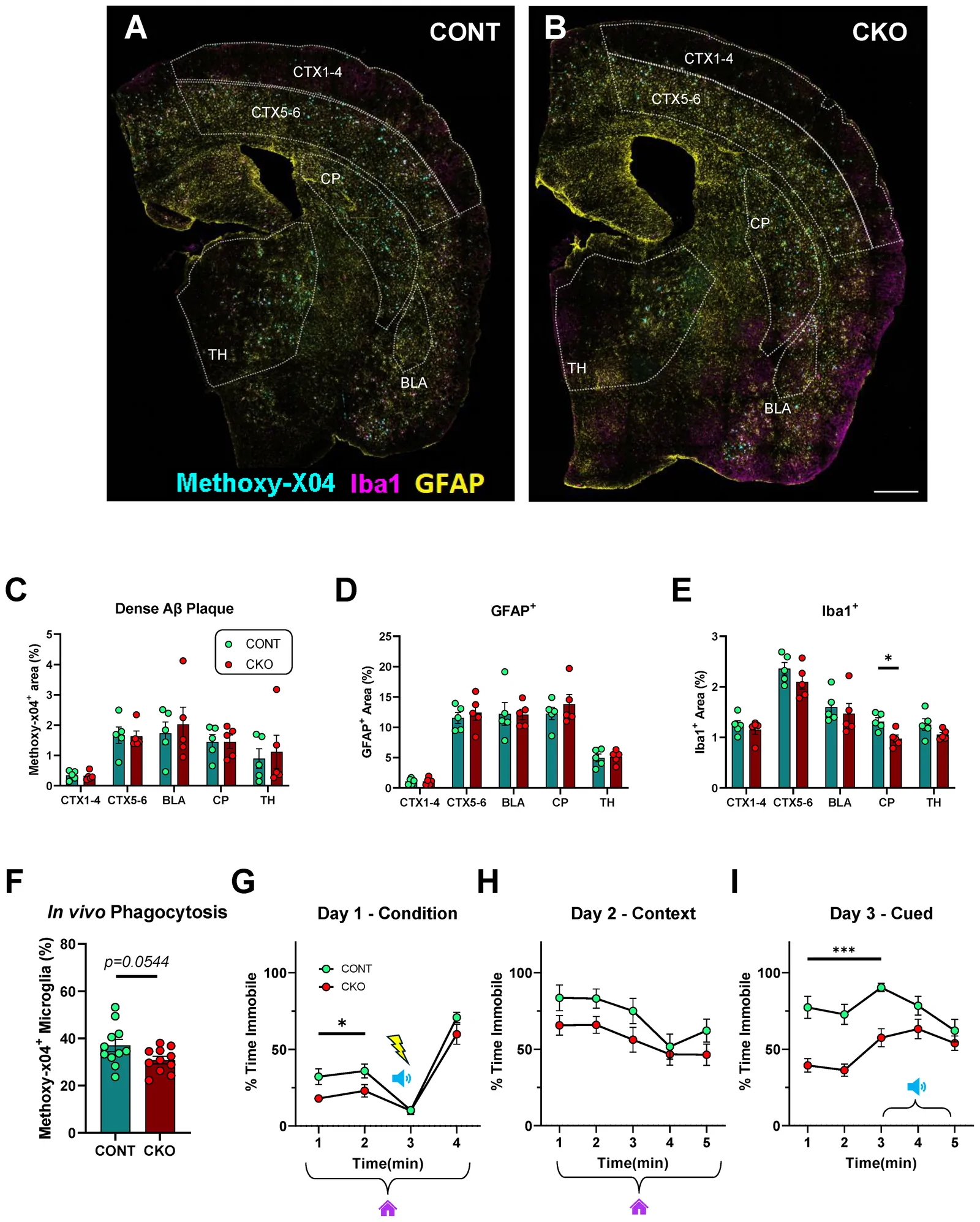
Evaluation of pathology, Aβ phagocytosis, and fear conditioning using 16-mo old mice. **(A, B)** IF detection of Methoxy-X04 (Aβ plaques), Iba1 and GFAP using 16-mo old CONT **(A)** or CKO **(B)** mice. Outlined regions are CTX1-4 (neocortex layer 1-4), CTX 5-6 (neocortex layer 5-6), CP (caudoputamen), BLA (basal lateral amygdala), and TH (thalamus). Scale bar, 200μm. **(C–E)** Quantification of areas of the fluorescent signals of Methoxy-X04 **(C)**, GFAP **(D)** and Iba1 **(E)** in the indicated areas. **(F)** *In vivo* phagocytosis of Aβ by microglia using16-mo old 5xFAD CONT and CKO mice. Each data point denotes a value from a mouse. Mean ± SEM. *p<0.05 by two-tailed unpaired Student’s t-test. **(G–I)** Fear Conditioning (FC) test of 16-mo old mice. The percentage of time freezing is shown during conditioning on day 1 **(G)**, contextual test on day 2 **(H)**, and cued test on day3, with the CS presented from 3 to 5 minutes **(I)**. Each data point denotes an average value ± SEM of mice in CONT (*n=9*) and CKO (*n=12*) groups. Repeated-measures ANOVA followed by Bonferroni’s multiple comparison tests were used to determine *post-hoc* differences among groups. ***p < 0.001..

Given the behavioral differences observed at 9- and 13-mo (**Figure 5**; **Supplementary Figure 6**), we examined whether CONT and CKO mice exhibited immunological or histological differences in aged mice. At 13- and 16-mo, the numbers of brain-infiltrating leukocytes were comparable between CONT and CKO groups, except for a small but significant increase in the proportion of infiltrated myeloid cells and B cell counts in 13-mo-old CKO mice (**Supplementary Figures 8**, **9A–H**). No changes were found in neutrophils and CD4^+^ T cells in 13- and 16-mo old mice, although they were previously altered at 9-mo (**Figures 4E, G**). Due to widespread Aβ accumulation at 16-mo, we analyzed multiple brain regions, including cortical layers (CTX 1–4 and 5-6), basolateral amygdala (BLA), caudoputamen (CP), and thalamus (TH) (**Figures 6A, B**). No significant differences were observed in dense Aβ plaque burden or GFAP-positive areas between groups (**Figures 6C, D**). A slight increase in the Iba-1^+^ signal was detected in the CP of CONT mice, suggesting a modest increase in microglia (**Figure 6E**). *In vivo* Aβ phagocytosis was also unchanged between groups at 16-mo old (**Figure 6F**). In summary, no major histological and immune cell recruitment were identified at 13 and 16 months.

### 3.11 Microglial dectin-1 promotes emotional responses in aged mice at 16-mo

To evaluate emotional memory and baseline anxiety, we conducted fear conditioning on 16-mo old mice (25). On day 1, mice explored the conditioning chamber for 3 minutes before presentation of a 30-second tone (Conditioned stimulus, CS) paired with a 0.4 mA foot-shock (Unconditioned stimulus, UCS) in the final 2 seconds of conditioning (**Figure 6G**), and mice remained in the chamber for 30 seconds. On day 2 (context test), mice were returned to the conditioning chamber without CS or UCS (**Figure 6H**). On day 3 (cued test), they were placed into a novel chamber for 3 minutes without the CS and UCS; this was followed with continual CS for 2 minutes after entry (**Figure 6I**).

On day 1, before conditioning, CONT mice showed significantly more freezing than CKO mice, indicating heightened baseline freezing in CONT mice (**Figure 6G**). This enhanced emotionality was evident—but not statistically significant—on day 2 during the early part of the context test (**Figure 6H**). On day 3 of cued fear testing, CONT mice showed significantly more freezing than CKO mice during the pre-CS period (0–3 min) in the neutral test chamber (**Figure 6I**). This difference may reflect greater generalization of conditioned fear in CONT mice. During the CS period (3–5 min), freezing increased in both groups and gradually converged, with similar freezing levels by the end of the period. Thus, the group difference was most evident before CS presentation rather than being sustained throughout the cued period. The two groups showed comparable foot-shock sensitivity (**Supplementary Figure 10**), indicating that their different freezing responses were unlikely to result from differences in shock perception. Collectively, these results suggest microglial dectin-1 contributes to heightened emotional responses in aged mice.

## 4 Discussion

In this study, we used microglia-specific dectin-1 CKO mice and demonstrated that microglial dectin-1 exerted either beneficial or detrimental effects, depending on age. Previous studies reported beneficial outcomes from dectin-1 agonist stimulation in the context of activating SYK, a signaling molecule downstream of dectin-1 (21, 22, 28). Ennerfelt et al. showed that bilateral intra-hippocampal injections of a dectin-1 agonist, pustulan, into 10-week old 5xFAD mice decreased total Aβ load detected by the D54D2 antibody in the hippocampus in a SYK-dependent manner (21). Wang et al. used a different approach, administering intraperitoneal injections of a dectin-1 agonistic antibody, and demonstrated enhanced Aβ phagocytosis and microglial activation (22). This study was performed in *TREM2^R47H^-5xFAD* mice, which carry a hypofunctional human TREM2 variant with impaired DAM signature acquisition. The results indicated that SYK activation via dectin-1 stimulation rescued the activation of hyporeactive microglia (22). Mangalmurti et al. used 5-mo old 5xFAD mice without microglial dectin-1, comparable to our CKO and showed doubled plaque burden, worsened neuritic dystrophy, and altered neuronal health gene expression, but had little effect on microglial activation, plaque engulfment, or DAM acquisition (28). Together, these reports demonstrated the beneficial effects of microglial dectin-1. However, it remained unclear how microglial dectin-1 affected behavior across different age groups. Therefore, we evaluated this in the present study.

Our histology data using 6-mo old mice also showed a beneficial role reported previously (21, 22, 28), but, despite reduced Aβ, we observed no significant differences in NORM or MWM at this age—tests that were not assessed in any other studies. Interestingly, at 9 months, the role of microglial dectin-1 became detrimental to behavioral performance with minimal detrimental effects on histological readouts.

Several factors may explain the age-dependent effects of dectin-1. First, the effects of microglial dectin-1 may differ across disease stages. For instance, Ennerfelt et al. (21) used 10-week old mice, so it remains to be determined whether pustulan injections would still be beneficial in older animals. Second, although the age of mice in the dectin-1 antibody study by Wang et al. is unknown (22), systemic versus microglia-specific dectin-1 stimulation may yield different outcomes. Third, using an EAE model, we previously demonstrated that dectin-1 cytoplasmic signaling bifurcates into CARD9-dependent and CARD9-independent pathways, driving proinflammatory or neuroprotective gene expression (9). Presently, the determinants of pathway preference remain unknown; therefore, it may be informative to evaluate whether switching between these pathways occurs in an age-dependent manner. Fourth, ligand type may be critical for the roles of age-dependent microglial dectin-1: Curdlan induces proinflammatory cytokines, such as IL-6 and TNF via SYK (6), whereas dectin-1 recognition of annexins on apoptotic cells promotes immune tolerance through selective SYK phosphorylation at Y348 and Y352 (10). Increasing evidence shows that dectin-1 signaling outcomes during sterile CNS inflammation can be either beneficial or detrimental, depending on context (7). With aging and disease progression, brain microenvironments may alter both the availability and nature of endogenous dectin-1 ligands, potentially shifting pathway utilization toward harmful or protective outcomes.

In 6-mo old mice, CONT mice showed fewer Aβ plaques in the subiculum, although no statistical difference emerged between CKO and CONT groups in behavior. Our data also indicated that microglial dectin-1 altered the spatial distribution of microglia and astrocytes around plaques in the cortex. (The cortex was studied in the cellular distribution analysis because dense plaques and glial signals prevented reliable spatial analysis in the subiculum.) The cortical glial distribution changes do not explain the subiculum plaque phenotype but may represent a separate, region-specific effect of microglial dectin-1.

At 9-mo of age, we did not observe apparent histological differences except that the hilus of the CONT mice showed the higher diffuse-to-dense Aβ plaque ratio than CKO mice. A higher diffuse-to-dense Aβ plaque ratio is interpreted as unfavorable, especially when assessing microglial plaque compaction. Of note, the age of 9-mo is when the age-dependent switch became apparent in microglial dectin-1’s roles. At 9- and 13-mo old mice, negative effects of microglial dectin-1 in cognition were identified. In MWM, female CONT mice required longer for spatial learning than female CKO mice, suggesting the detrimental role of microglial dectin-1. However, cognitive flexibility may benefit from the presence of dectin-1 in microglia, as CKO females retained a stronger preference for the original NE zone during reversal probe testing, consistent with reduced cognitive flexibility. Given that cognitive flexibility strongly associates with neurogenesis in the dentate gyrus (29–31) and that microglia support neurogenesis (32), it is possible that microglial dectin-1 supports neurogenesis in certain contexts while simultaneously exacerbating other cognitive domains. Notably, dectin-1/TLR2 stimulation promoted axon regeneration in optic nerve injury (17). We also demonstrated dectin-1-mediated neuroprotection during EAE (9).

At 16-mo, pathological differences between the CKO and CONT groups were difficult to detect, possibly due to the advanced disease stage. However, microglial dectin-1 was associated with increased freezing before conditioning and during the pre-CS period, as reflected by increased freezing of CONT mice before conditioning. This suggests that microglial dectin-1 may promote heightened emotional reactivity or generalized fear responses, even in the absence of explicit threats. If confirmed, this would position microglial dectin-1 as a potential target for modulating pathological anxiety or emotional dysregulation in neurodegenerative disease.

The behavioral differences at 13 and 16 months were not accompanied by changes in the histological or immune-cell measurements examined here. However, our analysis focused on selected endpoints and may not have detected subtle, region-specific, or functional changes, including alterations in synapses, neuronal circuits, soluble Aβ, or glial states. Future studies will be needed to identify the mechanisms linking microglial dectin-1 to these late-stage behavioral effects.

From an immunological standpoint, CKO mice at 9-mo showed significantly more neutrophil brain infiltration, particularly neutrophils with lower Ly6G expression. If neutrophils are considered to be proinflammatory, this result would be paradoxical as CKO mice had better behavioral performances. However, Ly6G^lo^ neutrophils are particularly known to exert neuroprotective effects, such as promoting neuronal survival after traumatic brain injury (18).

Independently of whether these cells correspond to the previously described Ly6G^lo^ subset, neutrophils are also known to ameliorate EAE after the disease peak (33). Since our CKO mice lack microglial dectin-1, but not peripheral, neutrophils in CKO mice could still respond directly to dectin-1 ligands. The functional polarization of these infiltrating neutrophils warrants further investigation, as their presence may partially explain improved behavioral outcomes in older CKO mice.

In summary, our data reveal an age-dependent switch in microglial dectin-1 functions—from beneficial effects on Aβ pathology in mid-life to detrimental effects on cognition and anxiety-related behavior in later life. This duality underscores the importance of context—age, ligand type, signaling pathway utilization, and cellular environment—in shaping microglial dectin-1 outcomes. Therapeutic strategies aiming to target dectin-1 in Alzheimer’s disease will therefore need to consider disease stage, microenvironmental conditions, and potential effects on non-microglial immune populations. Future studies should define the ligand and signaling landscape of microglial dectin-1 across the disease course, with the goal of identifying windows where intervention could tilt its function toward sustained neuroprotection.

## Data Availability

The original contributions presented in the study are included in the article/**Supplementary Material**. Further inquiries can be directed to the corresponding author/s.

## Supplementary material

Supplementary Figure 1 Confirmation of dectin-1 conditional knockout in microglia. **(A)** Allele-specific PCR was performed across both 5’ and 3’ integration junction sites using the indicated primer pairs. The clone D9 was selected with the correct PCR amplicon sizes of 2,256 bp and 910 bp for the 5’ and 3’ sites, respectively. **(B, C)** Flow cytometry validation of dectin-1 expression. Rat anti-mouse dectin-1 (clone 2A11) antibody was used **(B)** to detect dectin-1 expression on Microglia (CD45^lo^CD11b^+^) and brain-infiltrated myeloid cells (CD45^hi^CD11b^+^) in CKO and CONT mice **(C)**. Data from four 9-mo old mice per group were shown. **(D)** Representative image demonstrating the absence of dectin-1 expression on Iba-1^+^ microglia in a 9-mo old CKO mouse, but not in a 9-mo old CONT mouse.

Supplementary Figure 2 Gating strategy used to analyze brain-infiltrated leukocytes. Brain cells were isolated and stained for flow cytometry as described in Methods. Single cells were first gated, and CD45^+^ live cells were selected for further gating to microglia (MG), total myeloid cells, and total lymphocytes. Neutrophils were gated as Ly6G^+^ from myeloid cells. B cells, CD4^+^ and CD8^+^ T cells were identified within the lymphocyte gating.

Supplementary Figure 3 Histological analysis of 6-month-old mice. **(A)** Five regions, indicated with squares, in the cortex were used to quantify Methoxy-X04^+^ Aβ plaque areas and sizes. Scale bar, 300 μm. **(B)** Dense Aβ plaque areas were detected by Methoxy-X04 staining and quantitated for comparison. Percentages and areas of Methoxy-X04^+^ Aβ are shown. **(C, D)** Glial cell frequencies in arbitrary selected five areas of cortex. Percentages of Iba1-positive areas (C) and GFAP-positive areas **(D)** were quantified. Average astrocyte sizes were also evaluated (D). **(E, F)** Areas of Iba1^+^ cells (E) and GFAP^+^ cells and their average sizes (F) in the subiculum. A 350-μm diameter circle, which fits the entire subiculum, was evaluated. Each point denotes average values from five regions obtained from one mouse for cortex analysis. Each point denotes a value obtained from one mouse for subiculum analysis. Data are presented as mean ± SEM. Two-tailed unpaired Student’s t-test was used for statistical analysis.

Supplementary Figure 4 Behavioral evaluations of microglia-specific dectin-1 deletion in 6-mo old mice. **(A)** Novel Object Recognition Memory (NORM) tests performed with CONT and CKO mice at 6 months of age. From the left to the right, panels indicate preference for novel object (A value of 1 complete preference for the novel object, whereas 0 indixates equal exploration of the novel and familiar objects), time spent with both objects (sec), numbers of contact with objects, and distance traveled per 5-min as locomotor activity. **(B)** Morris Water Maze (MWM) test using 6-mo old mice. Swim distance (cm) and escape latency (swim time, sec) were measured across training days in control (CONT, green) and conditional knockout (CKO, red) mice. During the acquisition phase (Days 1–6), both groups showed progressive decreases in swim distance and latency, indicating learning of the platform location. During the reversal phase (Days 7–14), when the platform location was changed, both groups again improved over time. CONT, *n=9*; CKO, *n=12*. Each data point denotes a value from one mouse. Data are presented as mean ± SEM. These data were analyzed by repeated-measures ANOVA; no significant differences were observed between CONT and CKO groups.

Supplementary Figure 5 Histological and flow cytometry analysis of 9-mo old mice. **(A, B)** Proportions of diffuse and dense Aβ plaque area were evaluated using immunofluorescent detection with the 82E1 Aβ antibody and Methoxy-X04, respectively (A). Diffuse-to-dense Aβ area ratio were evaluated (B). The dentate gyrus (DG) was used for evaluation. **(C)** Proportion of Iba1-positive and GFAP-positive areas in DG. **(D)** Similar analysis to (A) but focused on the hilus in the DG. **(E, F)** Total B cell (E) and CD8^+^ T cell (F) counts and proportions among CD45^+^ cells, from the flow cytometry data shown in **Figure 4**. Each point denotes a value obtained from one mouse. Data are presented as mean ± SEM. Two-tailed unpaired Student’s t-test was used; no significant differences were observed between CONT and CKO groups.

Supplementary Figure 6 NORM tests of 9- and 13-mo old mice. **(A, B)** Control for NORM tests. In 9-mo old mice (A) and 13-mo old mice (B), time spent with objects, frequencies of object contacts, and distance traveled within the test arena are shown. Data are presented as means ± SEM. Repeated-measures ANOVA with Bonferroni corrections. **(C–F)** NORM performance of female (C, D) and male (E, F) mice at indicated ages. The same cohort was used for the analysis. STM (C, E) and LTM (D, F) data are shown. For the data of 6-mo old mice, the dataset presented in **Supplementary Figure 4A** is shown again in (C–F) for the purpose of the side-to-side comparison with the data of 9- and 13-mo old mice. Each data point denotes a value from one mouse. CONT, *n=9*; CKO, *n=12*. Data are presented as mean ± SEM. Repeated-measures ANOVA with Bonferroni corrections. **, p < 0.05; ****, p < 0.0001.*

Supplementary Figure 7 MWM tests of 9-mo old mice. **(A, B)** Reversal trial data of MWM tests from 9-mo old male and female mice: swim distance (A) and swim time (B). CONT, *n=9*; CKO, *n=12*. **(C, D)** Female-only data extracted from (A, B). CONT, *n=5*; CKO, *n=6*. **(E–I)** Shown are male mice data of acquisition trial results for swim distance (E), swim time (F), probe test results (G), reversal swim distance (H), and reversal swim time (I). CONT, *n=4*; CKO, *n=6*. Each data point denotes an average value from one group (A–F) or from one mouse (G). Data are presented as mean ± SEM (G). Repeated-measures ANOVA was performed with no significant differences between CONT and CKO groups. **(J–L)** Fear Conditioning (FC) test of 9-mo old mice. Immobilized time percentages are indicated from the condition period with shock and tone at 3 min on day 1 (J), the context test by placing mice on day 2 into the same chamber used for conditioning (K), and the day 3 cued test with a tone played for 2 min starting at 3 min after placing the mice into a novel chamber (L). Data are presented as mean ± SEM for nine mice per group.

Supplementary Figure 8 Brain-infiltrated leukocytes in 13-mo old mice. **(A)** Total CD45^+^ cell counts, including microglia, from the brain. **(B–D)** Counts and proportions (per total CD45^+^ cells) myeloid cells are shown for microglia (B), total CD45^hi^ myeloid cells, not including microglia (C), neutrophils (D). **(E–H)** Counts and proportions (per total CD45^+^ cells) of total lymphoid cells (E), CD4^+^ T cells (F), B cells (G), and CD8^+^ T cells (H). Gating strategy is the same as shown in **Supplementary Figure 2**. Each data point denotes a value from one mouse.

Supplementary Figure 9 Brain-infiltrated leukocytes in 16-mo old mice. **(A)** Total CD45^+^ cell counts, including microglia, from the brain. **(B–D)** Counts and proportions (per total CD45^+^ cells) myeloid cells are shown for microglia (B), total CD45^hi^ myeloid cells, not including microglia (C), neutrophils (D). **(E–H)** Counts and proportions (per total CD45^+^ cells) of total lymphoid cells (E), CD4^+^ T cells (F), B cells (G), and CD8^+^ T cells (H). Gating strategy is the same as shown in **Supplementary Figure 2**. Each data point denotes a value from one mouse.

Supplementary Figure 10 Sensitivity to foot-shock for fear conditioning (FC) in 16-mo old mice. Shock reactivity was comparable between CONT and CKO groups. Each data point denotes a value for mice in the CONT (*n=6*) and CKO (*n=9*) groups. A scrambled foot-shock intensity of 0.4 mA was used for FC. Data are presented as means ± SEMs. No significant genotype differences; repeated measures ANOVA with Bonferroni corrections.
