# Supplementary material for: Microglial dectin-1/Clec7a in an alzheimer’s disease model: a double-edged sword across the lifespan
Source: Front Immunol. 2026 Sep 3;17:1864888. doi: 10.3389/fimmu.2026.1864888 (PMC13581708; doi:10.3389/fimmu.2026.1864888)

Supplementary Figure 1

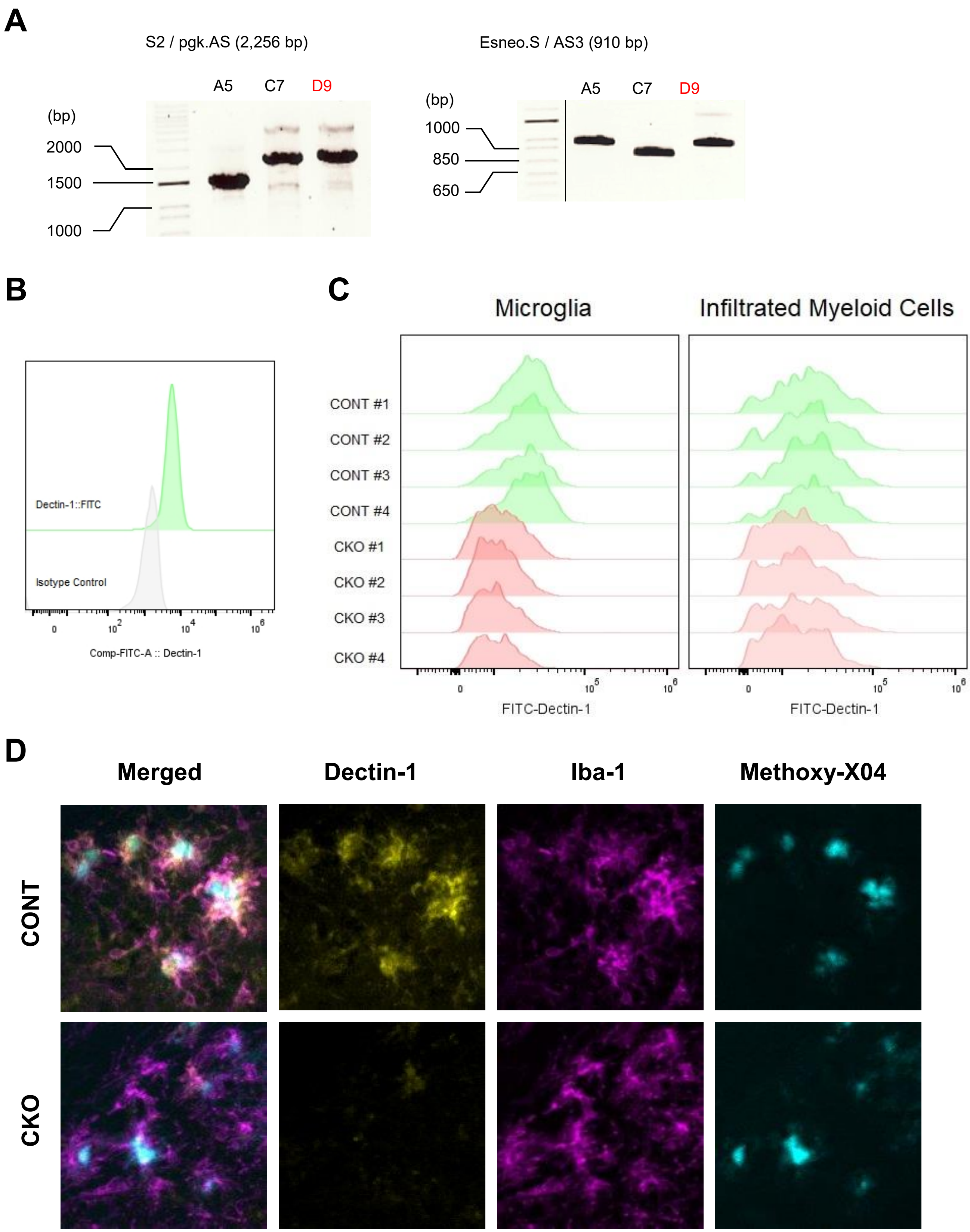

Supplementary Figure 2

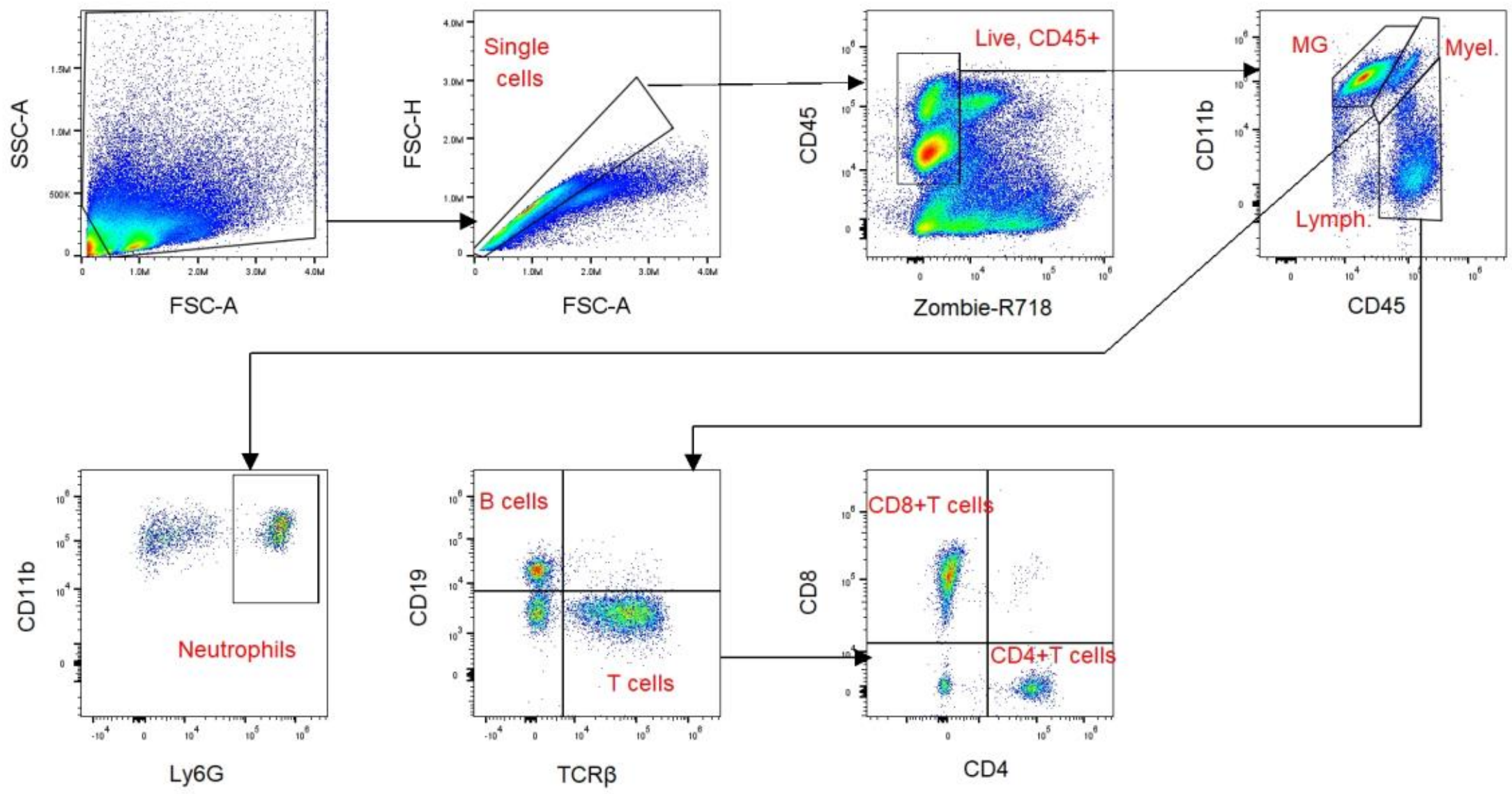

Supplementary Figure 3

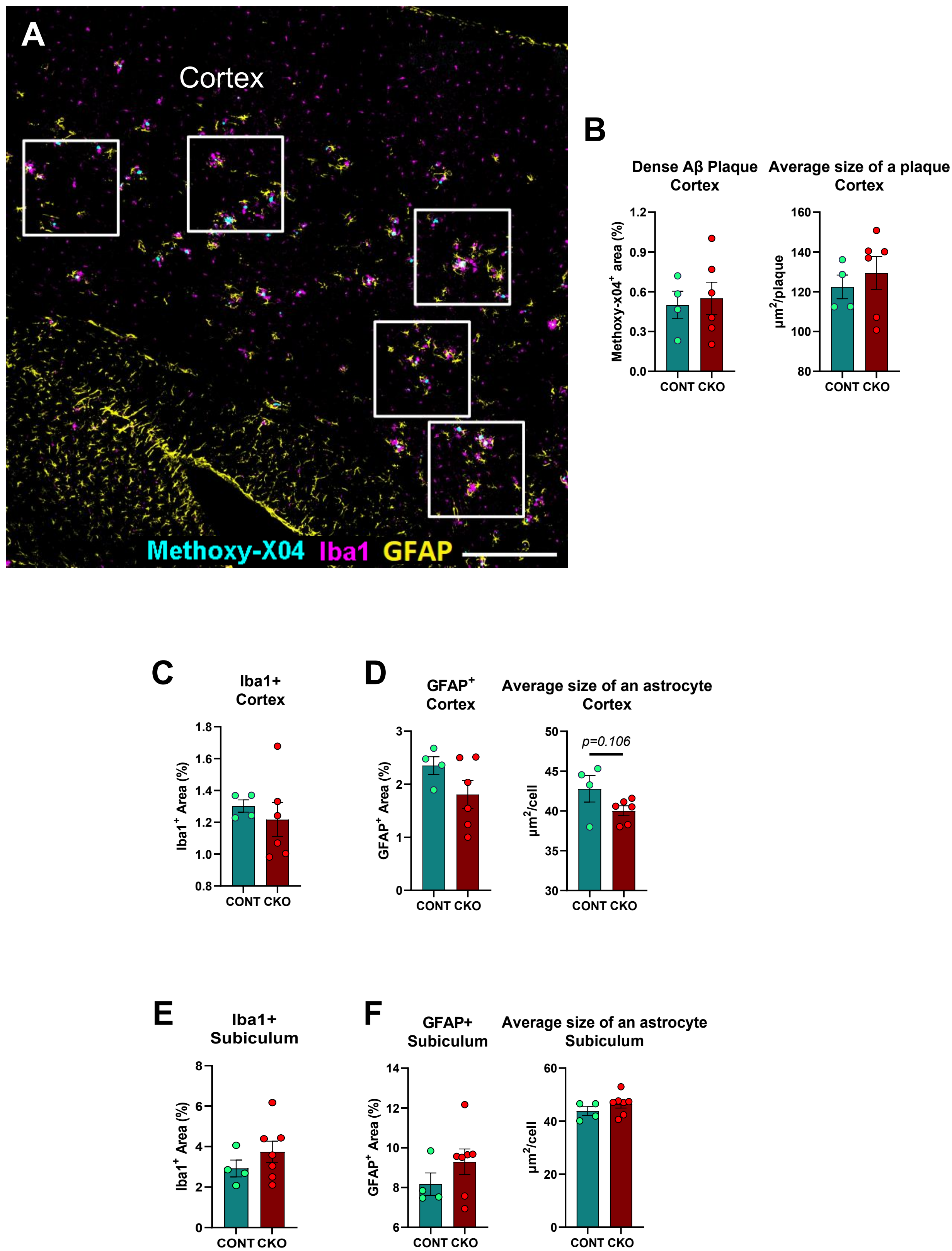

Supplementary Figure 4

A

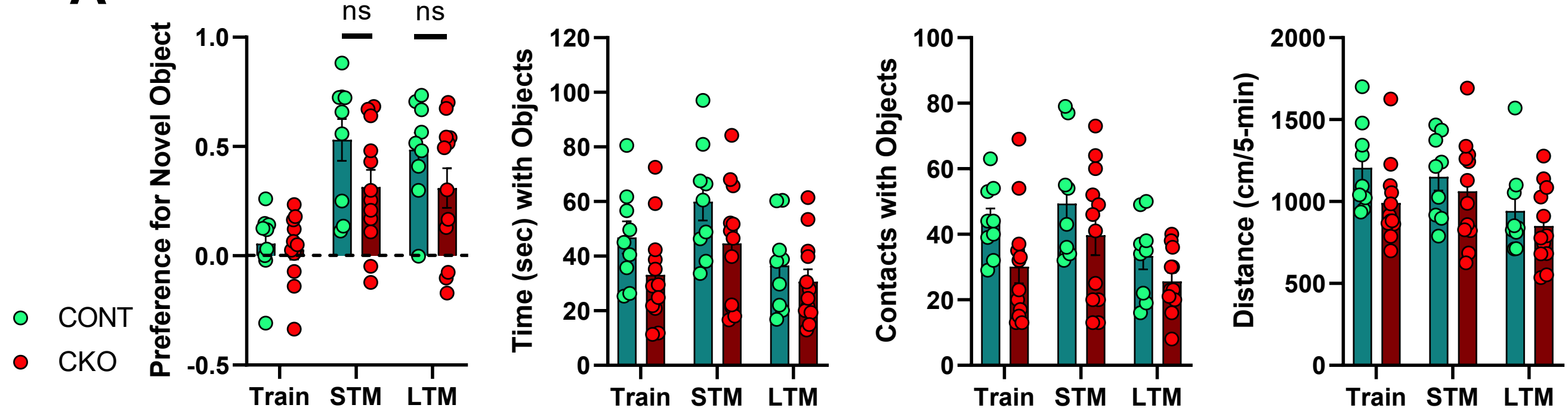

B

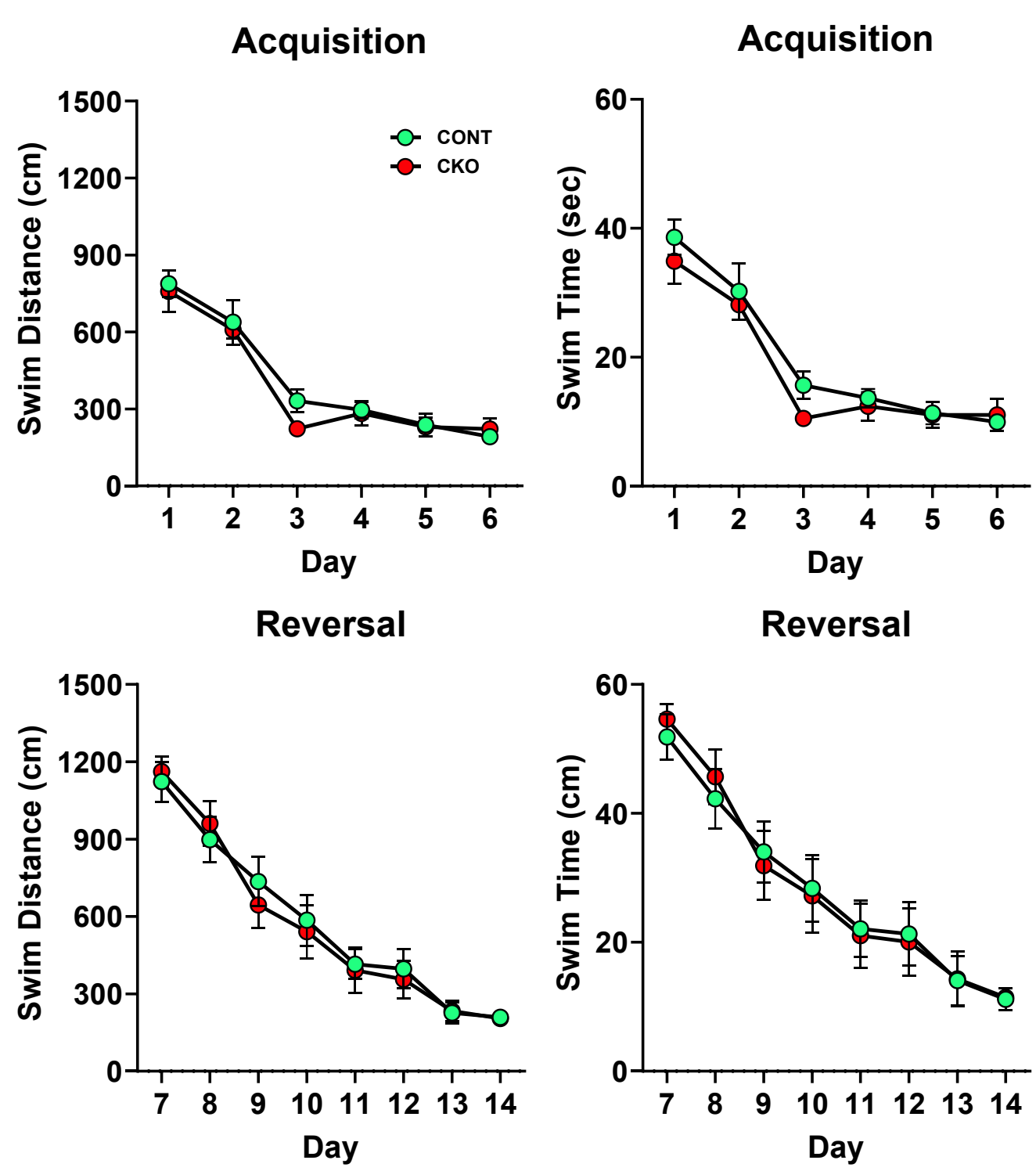

Supplementary Figure 5

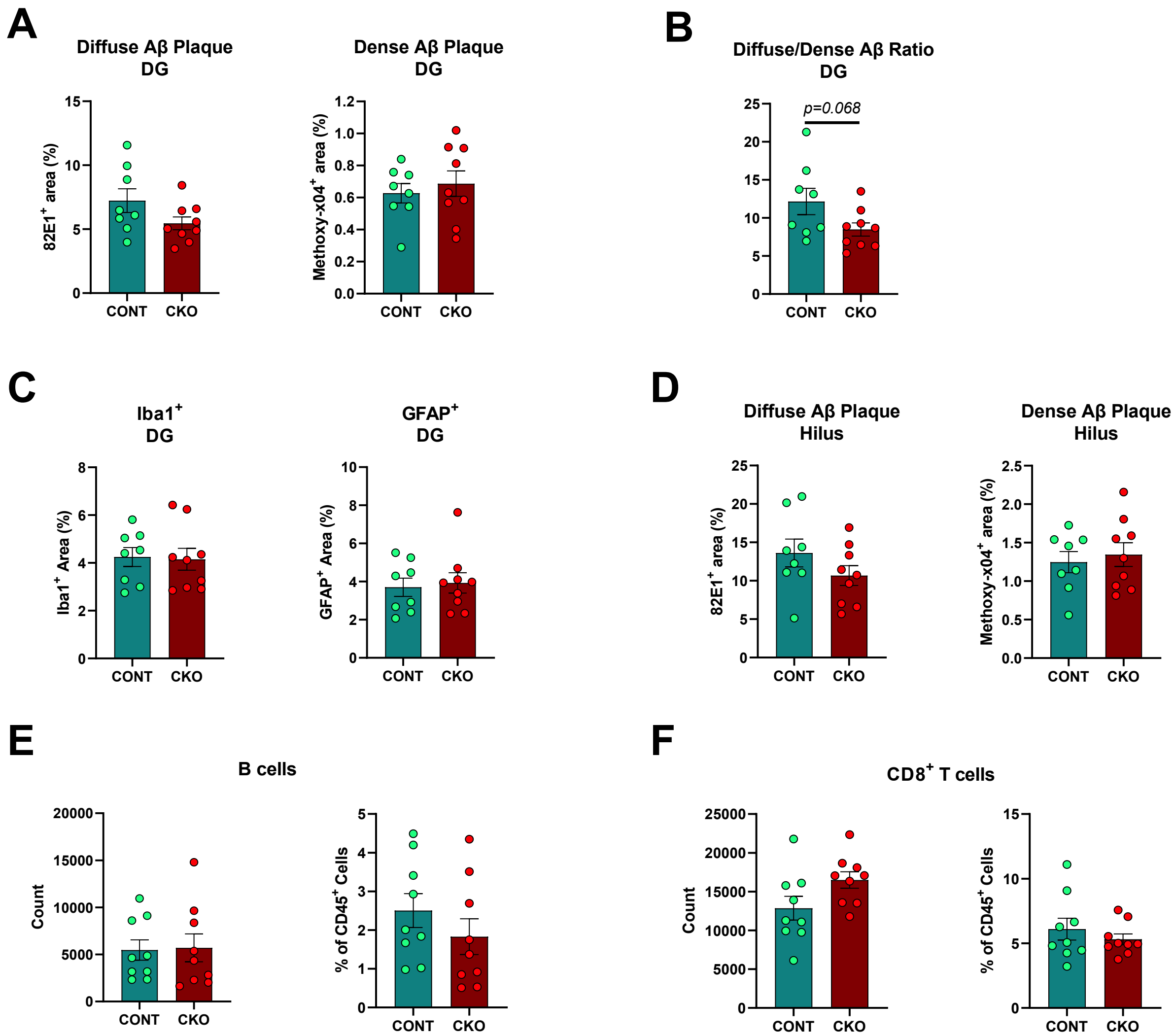

Supplementary Figure 6

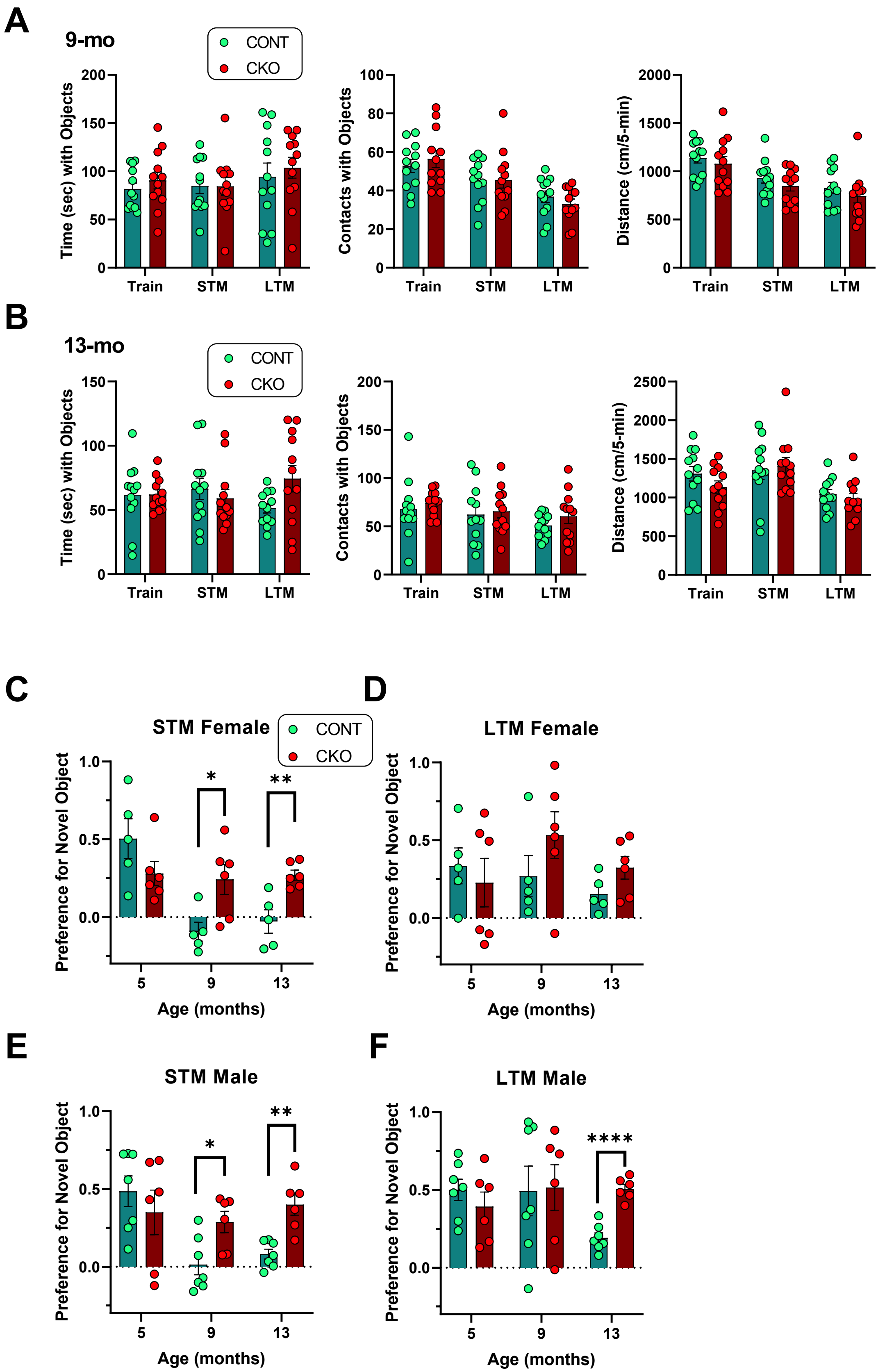

Supplementary Figure 7

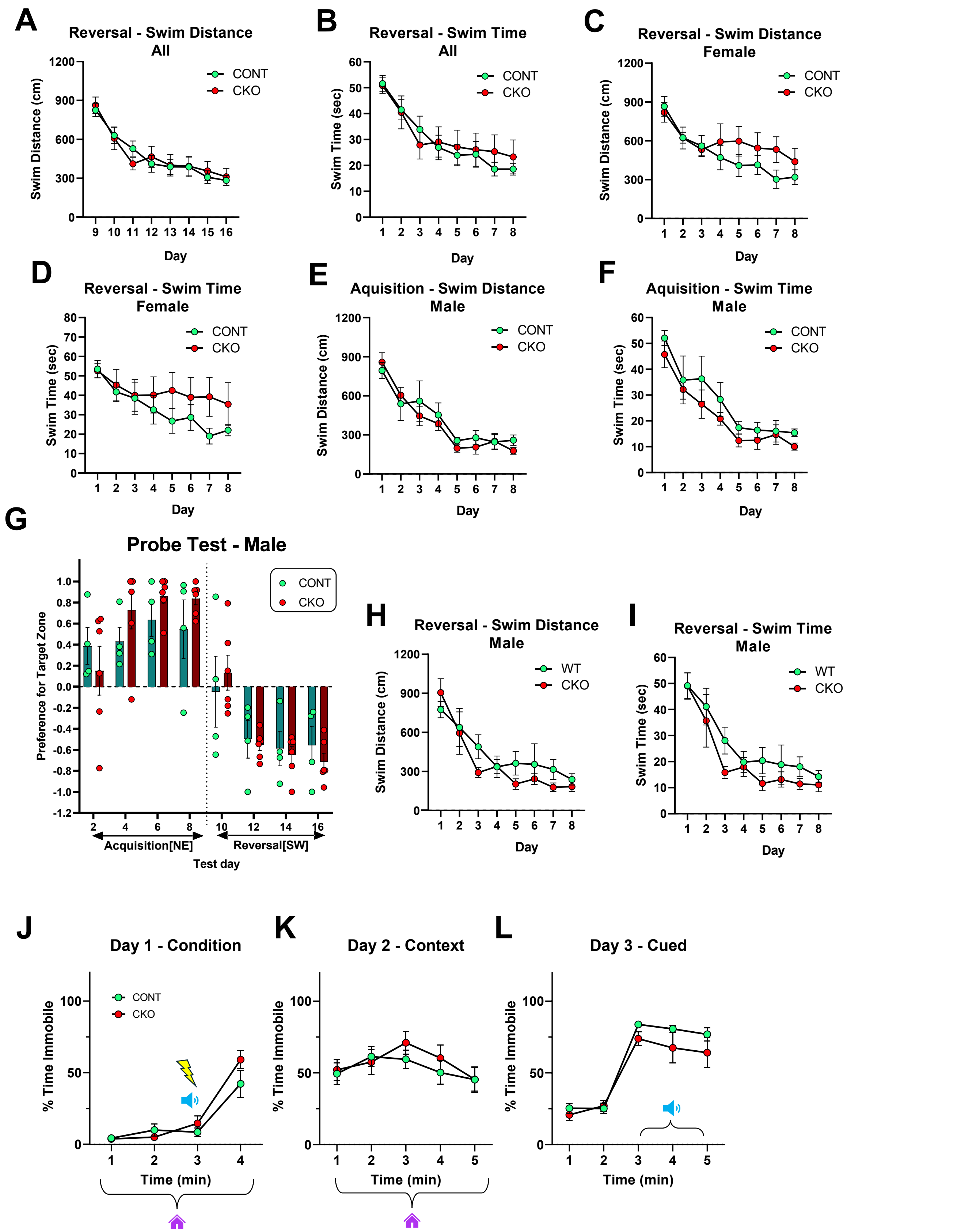

Supplementary Figure 8

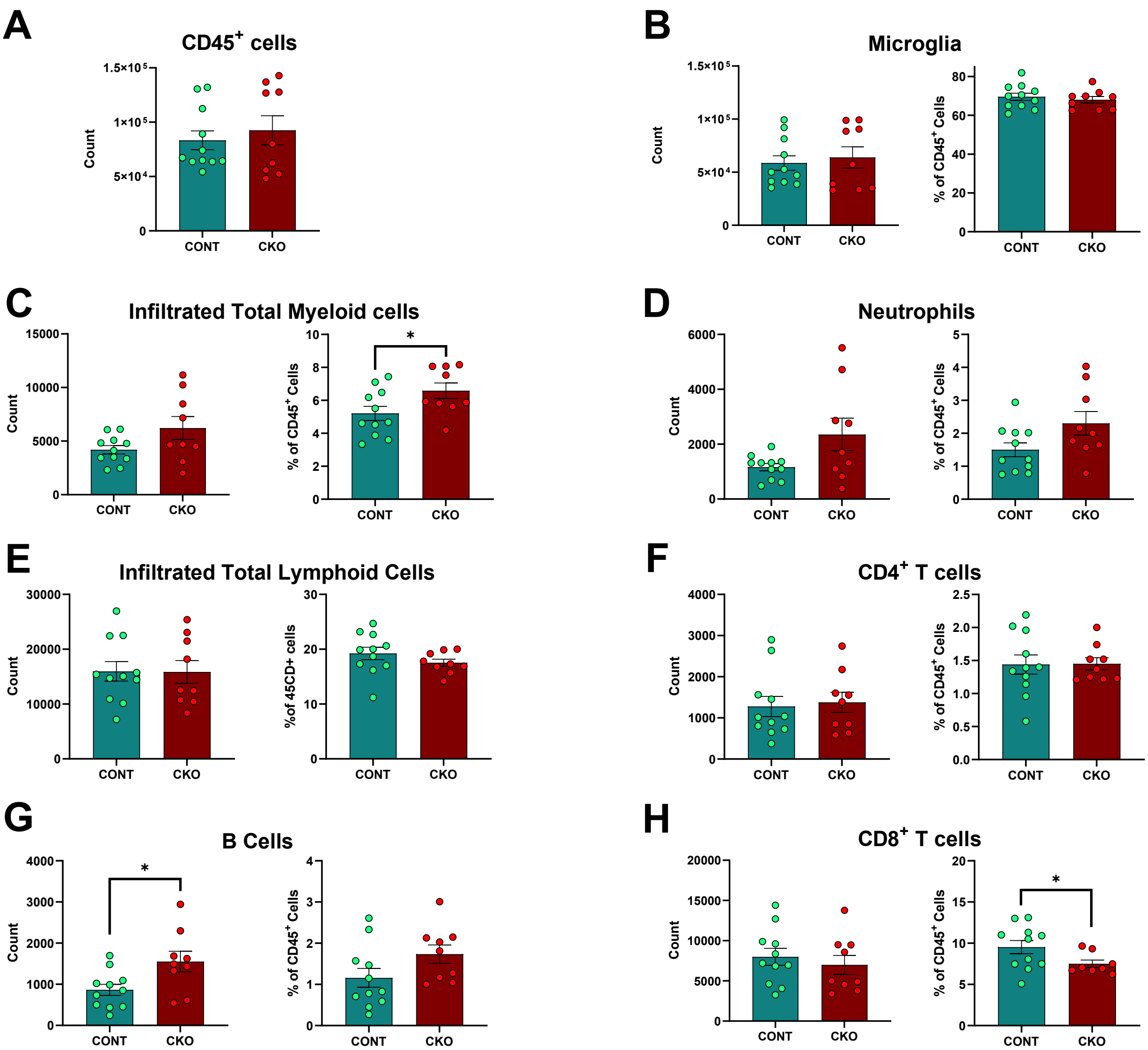

Supplemental Figure 9

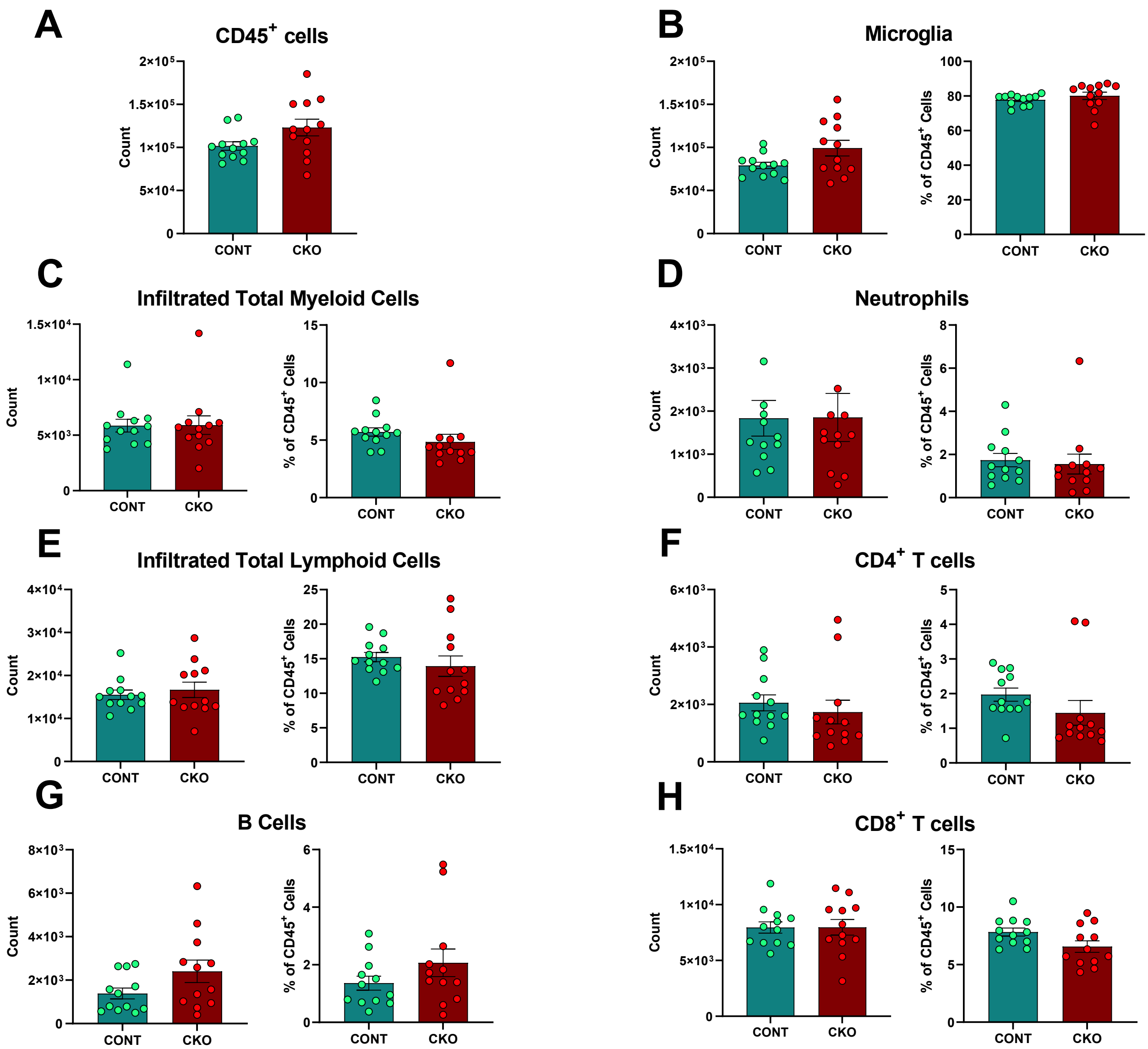

Supplementary Figure 10

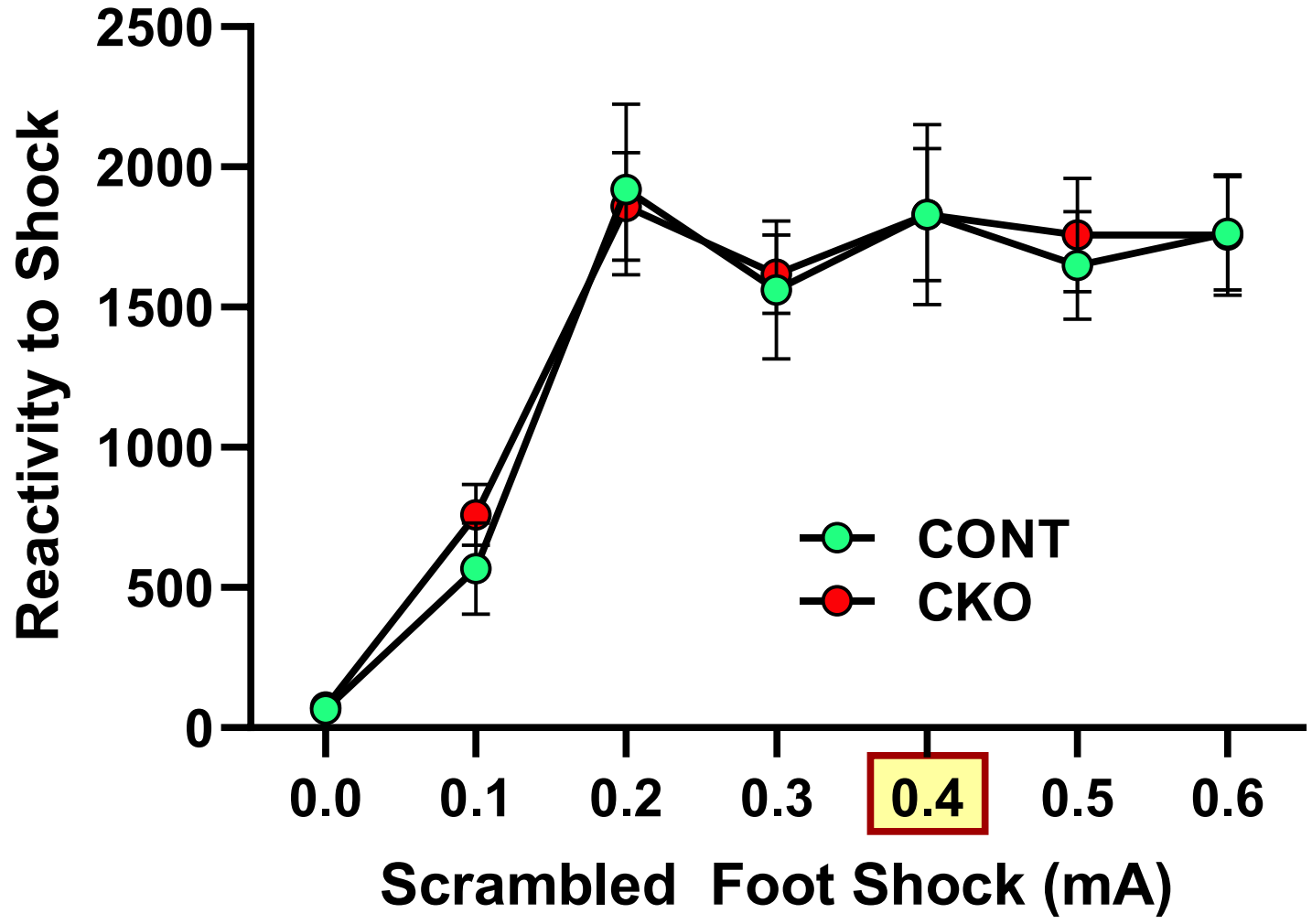

Supplement: Supplementary file 1 [file DataSheet1.pdf]
