# Supplementary material for: Microglial dectin-1/Clec7a in an alzheimer’s disease model: a double-edged sword across the lifespan
Source: Front Immunol. 2026 Sep 3;17:1864888. doi: 10.3389/fimmu.2026.1864888 (PMC13581708; doi:10.3389/fimmu.2026.1864888)
Supplement: Supplementary file 2 [file Table1.docx]

**Supplemental Table 1: List of DNA/RNA**

| **Name** | **Use** | **Sequence** |
| --- | --- | --- |
| sgRNA#1 | CRISPR Guide RNA#1 | GTCGCAAATAGAAAATGGCA |
| sgRNA#2 | CRISPR Guide RNA#2 | TGGTTCGGATGTTGTGGTAA |
| CLEC7A.Mock.S2 | ES cell genotyping Reaction 1 | GCTGTGTAATTTTCAAAGAGCTAGC |
| pgk.AS | ES cell genotyping Reaction 1 | AGACTGCCTTGGGAAAAGCGCCTC |
| Esneo.S | ES cell genotyping Reaction 2 | CTATCGCCTTCTTGACGAGTTCTTC |
| CLEC7A.Mock.AS3 | ES cell genotyping Reaction 2 | GTCTGAGCTAAGATGGAAAGTACG |
| Clec7a-fl-Neo-C7a.S | Clec7a^f/f^ Genotyping | TTTACATTCATCTGGGCTAGAGAG |
| Clec7a-fl-Neo-C7a.AS | Clec7a^f/f^ Genotyping | TCAAGCTTAGTGCCAAGTGCTACT |
| Cx3cr1-WT-F | Endogenous Cx3cr1(WT) Genotyping | AGCTCACGACTGCCTTCTTC |
| Cx3cr1-CreER-Common | Endogenous Cx3cr1(WT) Genotyping | ACGCCCAGACTAATGGTGAC |
| CreERT2-F1 | ERT2Cre Genotyping | GGCCCGCGCTGGAGTTTC |
| CreERT2-R1 | ERT2Cre Genotyping | GGGGGCTCAGCATCCAACAAG |
| 27367 | 5xFAD Genotyping | CGGGCCTCTTCGCTATTAC |
| 37598 | 5xFAD Genotyping | ACCCCCATGTCAGAGTTCCT |
| 37599 | 5xFAD Genotyping | TATACAACCTTGGGGGATGG |

**Supplemental Table 2: Reagents and Resources**

| **Reagent or Resource** | **Source** | **Identifier** |
| --- | --- | --- |
| **Antibodies** |  |  |
| Rat monoclonal anti-mouse I-A/I-E (clone M5/114.15.2) | BioLegend | Cat# 107608; RRID: AB_313323 |
| Armenian Hamster monoclonal anti-mouse CD11c (clone N418) | BioLegend | Cat# 117306; RRID: AB_313775 |
| Rat monoclonal anti-mouse Dectin-1 (clone 2A11) | BioRad | Cat# MCA2289FT; RRID: AB_324904 |
| Rat monoclonal anti-mouse CD45 (clone 30-F11) | BD Horizon | Cat# 564279; RRID: AB_2651134 |
| Rat monoclonal anti-mouse/human CD11b (clone M1/70) | BioLegend | Cat# 101257; RRID: AB_2565431 |
| Armenian Hamster monoclonal anti-mouse TCR β chain (clone H57-597) | BioLegend | Cat# 109210; RRID: AB_313433 |
| Rat monoclonal anti-mouse CD19 (clone 6D5) | BioLegend | Cat# 115545; RRID: AB_2562136 |
| Rat monoclonal anti-mouse Ly-6G (clone 1A8) | BioLegend | Cat# 127618; RRID: AB_1877261 |
| Rat monoclonal anti-mouse Ly-6C (clone HK1.4) | BioLegend | Cat# 128037; RRID: AB_2562630 |
| Rat monoclonal anti-mouse CD8a (clone 53-6.7) | BioLegend | Cat# 100750; RRID: AB_2562610 |
| Rat monoclonal anti-mouse CD4 (clone GK1.5) | BioLegend | Cat# 100414; RRID: AB_312699 |
| Rat monoclonal anti-mouse CD68 (clone FA-11) | BioLegend | Cat# 137004; RRID: AB_2044002 |
| Rat monoclonal anti-mouse CD16/32 (clone 93) | BioLegend | Cat# 101302; RRID: AB_312801 |
| Rat monoclonal anti-mouse CD45 (clone I3/2.3) | BioLegend | Cat# 147707; RRID: AB_2563539 |
| Rat monoclonal anti-mouse/human CD11b (clone M1/70) | BioLegend | Cat# 101208; RRID: AB_312791 |
| Chicken monoclonal anti-mouse/human Iba1 (clone Ch311H9) | Synaptic Systems | Cat# 234009; RRID: AB_2891282 |
| Mouse monoclonal anti-human N-terminal of Amyloidβ (clone 82E1) | IBL | Cat# 10323; RRID: AB_1630806 |
| Rabbit polyclonal anti-mouse GFAP | Abcam | Cat# ab7260; RRID: AB_305808 |
| Rat monoclonal anti-mouse Dectin-1 (clone R1-8g7) | InvivoGen | Cat# mabg-mdect; RRID: AB_2753143 |
| Goat polyclonal anti-chicken IgY (H+L) Secondary Ab | Thermo Fisher Scientific | Cat# A-21449; RRID: AB_2535866 |
| Donkey polyclonal anti-mouse IgG (H+L) cross-adsorbed Secondary Ab | Thermo Fisher Scientific | Cat# A10037; RRID: AB_11180865 |
| Donkey polyclonal anti-rabbit IgG (H+L) Secondary Ab | Molecular Probes | Cat# A-21206; RRID: AB_2535792 |
| Goat polyclonal anti-rat IgY (H+L) Secondary Ab | Molecular Probes | Cat# A-11081; RRID: AB_141738 |
| **Supplemental Table 2 *(continued)*** |  |  |
| **Transgenic Mouse** |  |  |
| 5xFAD | Jackson Laboratory | Cat# 034848-JAX**;**  RRID: MMRRC_034848-JAX |
| Cx3cr1^CreER^ knock-in/knock-out | Jackson Laboratory | Cat# 020940; RRID: IMSR_JAX:020940 |
|  |  |  |
| **Chemical and Reagents** |  |  |
| Zombie R718™ Fixable Viability Kit | BioLegend | Cat# 423115 |
| Methoxy-X04 | Cayman Chemical | Cat# 20476 |
| Tamoxifen | Cayman Chemical | Cat# 13258 |
| Collagenase D (from Clostridium histolyticum) | Roche | Cat# 11088866001 |
| DNase I, Bovine Pancreas | Millipore | Cat# 260913 |
| Heparin | Sagent Phar. | Cat# 25021-400-30 |
| Bovine Serum Albumin | Sigma-Aldrich | Cat# A7906-100G |
| Fetal Bovine Serum | GeminiBio | Cat# 100-106 |
| Percoll density gradient media | Cytiva | Cat# 17089101 |
| Paraformaldehyde | Sigma-Aldrich | Cat# 158127 |
| Tissue-Tek O.C.T. Compound | Sakura Finetek | Cat# 4583 |
| Triton X-100 | VWR | Cat# 0694-1L |
